# Signal processing and generation of bioactive nitric oxide in a model prototissue

**DOI:** 10.1038/s41467-022-32941-6

**Published:** 2022-09-06

**Authors:** Songyang Liu, Yanwen Zhang, Xiaoxiao He, Mei Li, Jin Huang, Xiaohai Yang, Kemin Wang, Stephen Mann, Jianbo Liu

**Affiliations:** 1 State Key Laboratory of Chemo/Biosensing and Chemometrics, College of Chemistry and Chemical Engineering, College of Biology, Key Laboratory for Bio-Nanotechnology and Molecular Engineering of Hunan Province, Hunan University, Changsha, 410082 People’s Republic of China; 2 Centre for Protolife Research, School of Chemistry and Max Planck-Bristol Centre for Minimal Biology, University of Bristol, Bristol, BS8 1TS UK; 3 School of Materials Science and Engineering, Shanghai Jiao Tong University, Shanghai, 200240 People’s Republic of China

**Keywords:** Synthetic biology, Networks and systems biology, Bioinspired materials, Origin of life

## Abstract

The design and construction of synthetic prototissues from integrated assemblies of artificial protocells is an important challenge for synthetic biology and bioengineering. Here we spatially segregate chemically communicating populations of enzyme-decorated phospholipid-enveloped polymer/DNA coacervate protocells in hydrogel modules to construct a tubular prototissue-like vessel capable of modulating the output of bioactive nitric oxide (NO). By decorating the protocells with glucose oxidase, horseradish peroxidase or catalase and arranging different modules concentrically, a glucose/hydroxyurea dual input leads to logic-gate signal processing under reaction-diffusion conditions, which results in a distinct NO output in the internal lumen of the model prototissue. The NO output is exploited to inhibit platelet activation and blood clot formation in samples of plasma and whole blood located in the internal channel of the device, thereby demonstrating proof-of-concept use of the prototissue-like vessel for anticoagulation applications. Our results highlight opportunities for the development of spatially organized synthetic prototissue modules from assemblages of artificial protocells and provide a step towards the organization of biochemical processes in integrated micro-compartmentalized media, micro-reactor technology and soft functional materials.

## Abstract (web summary)

A challenge for synthetic biology is the design and construction of prototissue. Here, the authors spatially segregate layers of enzyme-decorated coacervate protocells as a model prototissue capable of chemical signal processing and modulating outputs of nitric oxide to inhibit blood clot formation.

## Introduction

Living tissues comprise interconnected populations of specialized cells that operate collectively to perform specific functions. Establishing models of living tissues in synthetic constructs (prototissues)^1–3^ is a challenging endeavour, requiring the spatiotemporal ordering and integration of cytomimetic agents (protocells)^4–7^ to produce modules capable of chemical information processing and collective outputs. Synthetic protocells have been prepared using lipid and polymer vesicles^8–11^, semi-permeable proteinosomes and polysaccharidosomes^12,13^, inorganic colloidosomes^14^, organoclay/DNA capsules^15^, water-in-oil microemulsions^16^, and membrane-free coacervate microdroplets^17–22^. The latter have been coated with polymers^23,24^, polyoxometalates^25,26^, fatty acids^27^, phospholipids^28,29^ and surface-active colloids including derivatized proteins^30^, red blood cell membrane fragments^31^, yeast cell membrane components^32^ and jammed arrays of gold nanoparticle surfactants^33^ to produce membranized coacervate droplets consisting of a semi-permeable outer shell and molecularly crowded interior.

As a step towards the construction of 3D functional prototissues, various protocell models have been used as building blocks for the assembly of integrated three-dimensional (3D) architectures that exhibit coordinated behaviours such as signal communication, chemomechanical deformation and enzyme-mediated metabolism. A key strategy is to employ protocell-protocell contact-dependent interactions as the basis for prototissue assembly. For example, vesicle prototissues with spheroidal or sheet-like morphologies and controllable adhesion strengths and compaction have been prepared using membrane-mediated interactions involving streptavidin-biotin recognition or single-strand DNA complementarity^34^. Alternatively, externally applied acoustic or magnetic fields have been used to physically manipulate giant unilamellar lipid vesicles into localized prototissue architectures with coded configurations and microscale patterns^35–37^. In other studies, tissue-like enzymatically active spheroids that exhibit coordinated contractibility and mechanochemical transduction have been fabricated by the programmed chemical ligation of mixed populations of bio-orthogonally activated proteinosomes^38–40^. Furthermore, 3D printing has been used to prepare functional synthetic tissues based on contact-dependent interactions in networks of hemi-fused lipid-stabilized water-in-oil emulsion droplets^41,42^. The networks exhibit controlled mass transport and mutual communication via membrane pores to generate chemical and electrochemical circuitry and light-induced gene expression^43–45^.

Based in part on the mimicking of extracellular matrix/living cell interactions, synthetic prototissues have also been constructed by immobilizing populations of artificial protocells in soft viscoelastic aqueous media such as polysaccharide hydrogels. For example, millimetre-sized emulsion droplets have been stabilized by entrapment in an alginate matrix^46,47^, and enzyme-active proteinosomes immobilized in helical hydrogel filaments to implement signal-induced movement and protocell-mediated micro-actuation^48^. Membrane-less coacervate droplets have been captured in single hydrogels by self-immobilization^49^ or incarcerated in different hydrogel modules to produce a linear modular micro-reactor capable of a photocatalytic/peroxidation cascade reaction under non-equilibrium flow conditions^50^.

Here, we describe a prototissue model based on the hydrogel immobilization and spatial segregation of catalytically active coacervate vesicles. The coacervate vesicles are employed as an integrated cytomimetic model^28^ and re-designed as a stable biochemical reaction platform for the construction of a model prototissue with potential real-world applications. In general, coacervate vesicles offer novel opportunities in protocell science as in principle enzymes can be attached to the lipid outer surface to generate molecularly crowded micro-compartmentalized objects capable of membrane-mediated catalysis. Moreover, by attaching enzymes specifically to the coacervate membrane rather than using diffusive free biomolecules, prototissue-like materials with internal organization and functional stability can be generated using hydrogel immobilization.

Using this strategy, here we assemble a three-layer tubular prototissue-like vessel capable of modulating the output of bioactive nitric oxide (NO). For this, we use a concentric arrangement of agarose hydrogels to spatially immobilize and segregate chemically communicating populations of enzyme-decorated phospholipid-enveloped polymer/DNA coacervate vesicles. Communication between the different coacervate vesicles is achieved by decorating their outer membranes with hydrophobically modified glucose oxidase (GOx), horseradish peroxidase (HRP) or catalase (CAT), which are attached prior to loading the cell-like constructs into the hydrogels. The three different protocell-loaded hydrogels are assembled as contiguous concentric modules to produce a tubular prototissue-like vessel that can implement logic-gate processing of a dual glucose/hydroxyurea input under reaction-diffusion conditions. We show that a distinct nitric oxide (NO) output can be produced in the internal lumen of the model prototissue by directional protocell-mediated processing using a specific spatial sequence of hydrogel modules. Under these conditions, a diffusive H_2_O_2_ signal generated by GOx/glucose activity in an outer hydrogel module activates HRP-decorated protocells in an adjacent middle layer. This results in a downstream reaction with hydroxyurea to produce NO, which diffuses into the central channel through an inner module comprising CAT-decorated coacervate vesicles. The latter depletes any residual and potentially toxic H_2_O_2_ to produce a distinct NO output in the central channel of the prototissue vessel. We use the NO output as a bioactive agent to inhibit platelet activation and blood clot formation in samples of plasma and whole blood located in the internal channel, thereby demonstrating proof-of-concept use of the model prototissue vessel for anticoagulation applications.

## Results

### Design and construction of a model prototissue tubular vessel

**Fig. 1:**
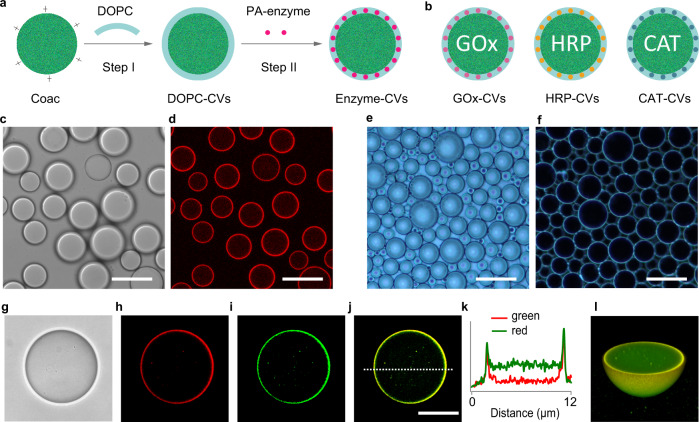
Assembly and characterization of enzyme-decorated coacervate vesicles. **a** Scheme showing design and construction of enzyme-decorated coacervate vesicles. Step I: a suspension of positively charged PDDA/DNA coacervate (Coac) micro-droplets is added to a DOPC/ethanol solution to produce phospholipid-based coacervate vesicles (DOPC-CVs). Step II: The DOPC-CVs are surface-decorated with palmitic acid-modified enzymes (PA-enzyme) to give molecularly crowded protocells with reactive outer membranes (Enzyme-CVs). **b** Types of enzyme-decorated coacervate vesicles produced; PA-glucose oxidase (GOx-CVs), PA-horseradish peroxidase (HRP-CVs) and PA-catalase (CAT-CVs). Labels marked inside the CVs (GOx, HRP and CAT) identify the different types of CVs; in each case, the PA-enzymes are attached to the outer membrane surface and not encapsulated. Bright field (**c**) and corresponding red fluorescence (**d**) images of undecorated DOPC-CVs (PDDA: DNA = 0.6: 1, w/w; DOPC: coacervate = 0.1: 1, w/w) stained with the lipophilic dye Dil (1,1^/^-dioctadecyl-3,3,3^/^,3^/^-tetramethyl indocarbocyanine perchlorate; red fluorescence, Ex: 549 nm, Em: 565 nm). Each coacervate droplet is encased in a continuous phospholipid membrane; scale bar, 10 μm. Optical phase contrast (**e**) and dark field (**f**) images of closely packed multiple layers of undecorated DOPC-CVs showing absence of coalescence. Optical (**g**), red fluorescence (**h**), green fluorescence (**i**), and merged red/green fluorescence (**j**) images of a single FITC-labelled PA-GOx-CV after staining with Dil. DOPC (red) and GOx (green) are co-located at the DOPC-CV membrane. A representative fluorescence intensity profile (**k**) corresponding to the dashed line in (**j**), and associated 3D reconstructed image (**l**), of single PA-GOx-decorated DOPC-CV. The DOPC membrane and enzyme surface attachment is uniform and continuous; 3 times each experiment was repeated independently with similar results (**c**–**j**, **l**). Scale bars (**g**–**j**), 5 μm.

To fabricate a model prototissue vessel capable of integrated chemical processing, we designed and constructed cell-like constructs in the form of enzyme-decorated membranized coacervate micro-droplets (Fig. 1a, b). To achieve this, positively charged (zeta potential, +13.2 ± 5.7 mV) membrane-less coacervate micro-droplets were prepared by associative liquid–liquid phase separation in aqueous mixtures of poly (diallyldimethyl ammonium chloride) (PDDA) and double-stranded DNA (Supplementary Fig. 1), and then coated in a continuous phospholipid membrane to produce stable dispersions of dioleoyl phosphatidylcholine (DOPC)-enveloped coacervate vesicles (DOPC-CVs). Bright field and fluorescence microscopy images showed discrete spherical droplets with a mean size of 20.0 ± 6.3 μm that revealed a bright red fluorescent outer shell when stained with the lipophilic lipid bilayer probe Dil (Fig. 1c, d). The DOPC-CVs were stable with respect to coalescence and could be closely packed and stacked into stable multi-layer arrangements, providing the possibility of high density packing in three-dimensional space (Fig. 1e, f). The presence of a distinct lipid-stained surface membrane was consistent with transmission electron microscopy (TEM) images of minimally sized DOPC-CVs extracted from the supernatant after centrifugation. TEM images of single DOPC-CVs showed an electron dense homogeneous coacervate matrix surrounded by a continuous membrane with an estimated thickness of 6.4 ± 0.5 nm, comparable to the thickness of a phospholipid bilayer (Supplementary Fig. 2). In contrast, no surface layer was observed by TEM for the membrane-free PDDA/DNA coacervate droplets (Supplementary Fig. 2).

Attachment of GOx, HRP or CAT to the outer membrane surface of the DOPC-CVs was achieved by increasing the lipophilicity of the enzymes by conjugation with palmitic acid (PA; hexadecanoic acid). Matrix-assisted laser desorption/ionization-time-of-flight mass spectrometry (MALDI-TOF MS) confirmed that the resulting PA-GOx, PA-HRP and PA-CAT nanoconjugates consisted on average of 1 to 6 hexadecanoic chains for each enzyme molecule (Supplementary Fig. 3). Addition of hydrophobically modified PA-GOx labelled with fluorescein-5-isothiocyanate (FITC) to a suspension of DOPC-CVs followed by treatment with a lipid stain produced protocells with red and green fluorescence co-localized specifically at the membrane (Fig. 1g–l). In contrast, native GOx (no PA-modification) did not bind to the phospholipid membrane of the DOPC-CVs (Supplementary Fig. 4). Similar observations were made for the binding of FITC-PA-HRP or FITC-PA-CAT to the DOPC-CVs (Supplementary Figs. 5, 6). In all cases, minimal levels of sequestered enzymes were observed in the coacervate core, consistent with a molecular weight cut-off for the lipid-coated coacervate droplets of approximately 6 kDa^28^. Molecular binding curves indicated that more than 82% of the enzymes were associated with the DOPC-CVs (Supplementary Figs. 7, 8).

**Fig. 2:**
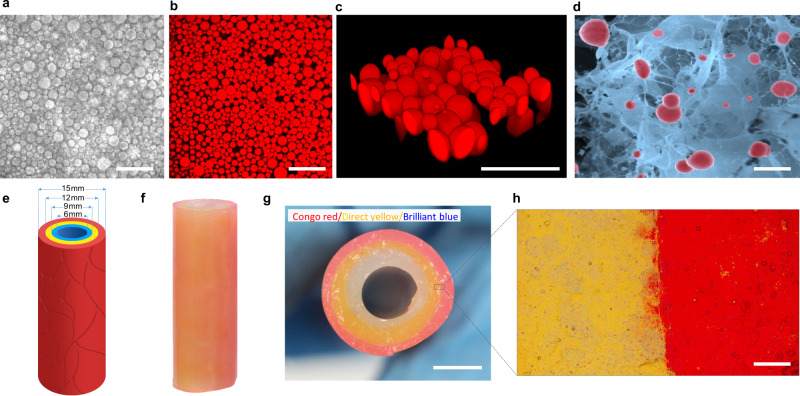
Design and construction of a tubular prototissue-like vessel. Optical microscopy bright image (**a**), fluorescence confocal image (**b**), and partial 3D reconstitution image (**c**) of a 1.0 wt% agarose hydrogel comprising a densely packed population of enzyme-decorated DOPC-CVs with sequestered sulforhodamine B (red fluorescence); scale bars; 50 μm. **d** SEM image of a lyophilized sample of hydrogel-immobilized DOPC-CVs showing intact CVs (false red coloration) physically attached to the 3D agarose network; scale bar, 30 μm. Graphic (**e**), and photographs viewed side-on (**f**) and end-on (**g**), showing tubular three-layer prototissue-like vessel with central 6 nm-wide channel. Spatial organization of the three protocell-loaded hydrogel modules (outer diameter, 15 mm) gives rise to concentrically arranged layers of immobilized DOPC-CVs decorated with different PA-enzymes (red, outer layer; yellow, middle layer; blue, inner layer) (**g**). Each hydrogel layer is 1.5 mm in thickness, with outer, middle and inner diameters of 15, 12, and 9 mm, respectively. Hydrogel modules shown in (**g**) are stained with Congo red (outer), Direct yellow 27 (middle), and Brilliant blue R (inner), respectively; scale bar, 5 mm. **h** Optical microscopy image of the interface between the middle (yellow) and outer (red) hydrogel layers. The imaged area is denoted by the rectangular box shown in (**g**); scale bar (**h**): 100 μm. Three times each experiment was repeated independently with similar results (**a**–**d**).

Having established a procedure for attaching lipophilic enzymes to the outer surface of the DOPC-CVs (enzyme-CVs), we sought to fabricate a series of protocell-based hydrogel modules as the basis for constructing a model prototissue. Each module consisted of a hydrogel that was loaded with a single population of immobilized enzyme-CVs. In each case, capture of approximately 2 × 10^6^ protocells per mL was achieved by slow cooling of an enzyme-CV/agarose aqueous suspension from 40 °C to room temperature. Optical and fluorescence microscopy images, 3D reconstructions and scanning electron micrographs of the loaded hydrogels showed colonies of intact protocells that were immobilized and spatially distributed throughout the agarose matrix (Fig. 2a–d). No significant changes in the shape or size of the enzyme-CVs were observed after immobilization. Rheometric analysis under 1% strain indicated that the storage modulus (*G*^*/*^) of the hydrogels increased with increasing agarose concentration to give protocell-loaded modules with relatively high stiffness (Supplementary Fig. 9). The *G*^*/*^ values were essentially unchanged by immobilization of the DOPC-CVs within the hydrogel matrix, indicating that the hydrogels were not destabilized when loaded with the protocells.

Based on the above observations, we assembled a self-supporting tubular prototissue-like vessel from a three-layer concentric arrangement of protocell-loaded hydrogel modules (Fig. 2e). For this, we implemented a stepwise construction process involving a three glass rod templates of decreasing diameters that were sequentially placed within a glass tube to generate tubular compartments that were infilled with a hot aqueous agarose suspension containing single populations of the enzyme-CVs, followed by in situ hydrogelation (Supplementary Fig. 10). The prototissue-like vessel was 100 and 15 mm in length and outer diameter, respectively, and contained a 6 mm-wide central channel (Fig. 2f, g). Each hydrogel layer was approximately 1.5 mm in thickness and bounded by distinct interfaces (Fig. 2h). A series of leakage experiments showed that release of the PA-conjugated enzymes from coacervate vesicle-containing hydrogels was strongly inhibited when compared with analogous experiments undertaken with hydrogels containing free enzymes and no coacervate vesicles (Supplementary Fig. 11), confirming efficient attachment of the hydrophobized enzymes to the outer membrane of the immobilized coacervate vesicles.

### Processing of nitric oxide generation in a model prototissue

**Fig. 3:**
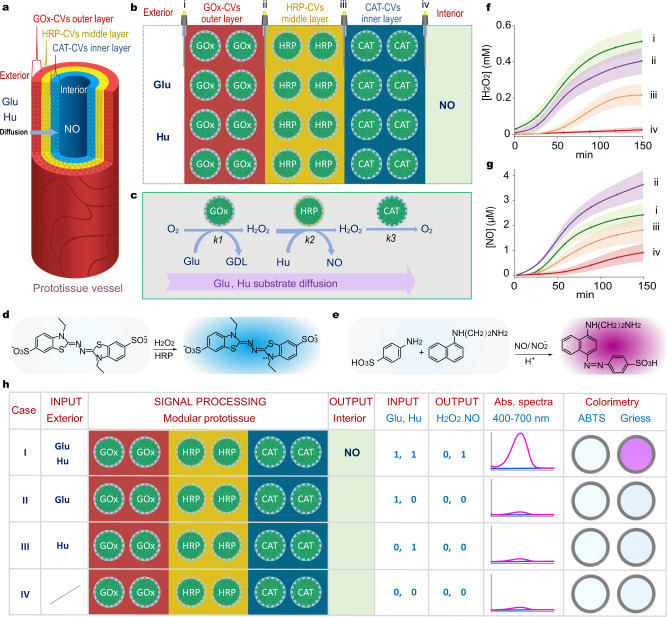
Signal processing of nitric oxide generation in a model prototissue. **a** Graphic showing tubular three-layer model prototissue vessel comprising immobilized populations of GOx-CVs, HRP-CVs or CAT-CVs in the outer, middle or inner hydrogel modules, respectively. **b** Graphic showing a cross-section of the three-layer modular prototissue-like vessel. Generation of H_2_O_2_ and NO were monitored at different positions (i–iv) using microelectrodes. **c** Reaction diagram for three-enzyme processing in the model prototissue vessel under reaction-diffusion conditions. H_2_O_2_ generation in the outer layer serves as a diffusive signal for HRP/Hu-mediated NO production in the middle layer. Excess H_2_O_2_ is removed by the CAT-CVs located in the inner layer adjacent to the lumen. Oxygen is consumed and produced in the outer and inner layer, respectively. *K1*, *k2*, and *k3* are the enzyme reaction rates for the GOx-CVs, HRP-CVs and CAT-CVs, respectively, immobilized within the different hydrogel modules. Reaction schemes for the colorimetric determinations of H_2_O_2_ (HRP-mediated ABTS oxidation, **d**) and NO (Griess reagent, **e**). Spatiotemporal distribution of H_2_O_2_ (**f**) and NO (**g**) at different positions ((i)-(iv), see (**b**)) in the prototissue-like vessel. Reaction conditions: Glu (50 mM); Hu (6 mM); GOx (GOx-CV outer layer, 0.2 mg mL^−1^); HRP (HRP-CV middle layer, 0.2 mg mL^−1^); CAT (CAT-CV inner layer, 0.1 mg mL^−1^). Data are presented as mean ± s.d. (*n* = 3 independent experiments). **h** Logic gate generation of NO in a three-layer tubular enzyme-CV modular prototissue-like vessel demonstrating AND gate processing. Only case I with a dual substrate input gives rise to a distinct NO output in the interior lumen. A H_2_O_2_ output in the lumen is rendered null by catalase activity in the inner layer. Representations of the corresponding absorption spectra (ABTS assay of H_2_O_2_ (blue); Griess assay of NO (purple) and colour changes for NO detection (Greiss assay, 540 nm, purple coloration) are also shown. A threshold absorption value above 0.04 at 540 nm (Greiss reagent, purple coloration with NO) was used as a verifiable (0,1) signal output. Reaction concentrations as given in (**f**, **g**).

Using the above procedures, we prepared a three-layered tubular model prototissue capable of generating the vasoactive and anti-coagulation agent, nitric oxide (NO), via a spatially confined protocell-mediated enzyme process operating under a substrate reaction-diffusion gradient. The concentrically arranged hydrogel modules contained single populations of immobilized PA-GOx-, PA-HRP- or PA-CAT-decorated DOPC-CVs arranged as the outer, middle, and inner layers, respectively, of the vessel (Fig. 3a). Assays undertaken on each module indicated that the PA-enzymes remained active when attached to the DOPC-CVs and immobilized in the agarose hydrogels (Supplementary Fig. 12). Given that HRP catalyses the conversion of hydroxyurea into NO in the presence of H_2_O_2_^51^, we reasoned that in situ production of H_2_O_2_ within the model prototissue could be used as a diffusive signal for generating NO within the central channel of the tubular vessel. To achieve this, glucose and hydroxyurea were added specifically to the exterior side of the model prototissue and then allowed to diffuse inwardly through the concentrically arranged hydrogels such that GOx-CV-mediated H_2_O_2_ production in the outer hydrogel module was subsequently processed downstream by HRP-CVs in the middle layer to produce NO. Under certain conditions, excess H_2_O_2_ was subsequently removed in the CAT-CV-containing inner layer, such that NO was the main output entering the central lumen (Fig. 3b). The amount of NO generated was governed by the interplay between the H_2_O_2_ and hydroxyurea reaction-diffusion gradients associated with the different enzyme reaction rates in the three different protocell/hydrogel modules (Fig. 3c and Supplementary Fig. 12). For example, increasing the loading of HRP-CVs in the middle hydrogel layer facilitated an increase of NO in the lumen, while decreasing the CAT-CV loading in the inner layer resulted in leakage of excess H_2_O_2_ into the central channel (Supplementary Figs. 13 and 14). Significantly, immobilization of the enzyme-CVs in a spatiotemporal sequence was critical for the efficient output of NO; in contrast, homogeneous mixing of the three different enzyme-decorated protocells in bulk solution or a uniform distribution of the three protocell types in the prototissue-like vessel produced minimal levels of H_2_O_2_ and NO under the same concentrations used in the tubular vessel (Supplementary Fig. 15). This was attributed to complete degradation of the H_2_O_2_ intermediate due to the considerably higher rate constant of catalase-mediated H_2_O_2_ decomposition compared with GOx-mediated H_2_O_2_ production and HRP-mediated peroxidation.

The spatiotemporal distributions of H_2_O_2_ and NO at different positions along the reaction-diffusion gradient of the prototissue-like vessel were monitored using selective microelectrodes, while colorimetric assays were used to determine H_2_O_2_ and NO concentrations in the central lumen (Fig. 3d, e). An extension in reaction time led to a gradual increase in the production of H_2_O_2_ and NO over 150 min (Fig. 3f, g). Concentrations of H_2_O_2_ were highest in the outer hydrogel module and progressively decreased in the middle and inner layers as the signalling molecule became depleted by downstream reactions with HRP/hydroxyurea and catalase, respectively (Fig. 3f). Minimal levels of H_2_O_2_ were detected in the aqueous solution trapped in the central channel, indicating that any excess H_2_O_2_ entering the inner layer was decomposed by the immobilized CAT-CVs. In contrast, after 150 min, the highest levels of NO were observed near to the boundary between the outer and middle hydrogel modules (Fig. 3g). NO was also detected in significant amounts at the exterior of the model prototissue vessel as well as in the inner layer and lumen, indicating non-directional diffusion of the output after formation in the middle layer. The initial rate of NO output in the central channel 30 minutes after the addition of glucose (50 mM) and hydroxyurea (6 mM) to the external medium was 2.4 nM min^−1^, resulting in the detection of NO concentrations in the interior lumen of up to 1.0 μM after 150 min. The production of NO was dependent on the glucose and hydroxyurea concentrations for a constant protocell number density, with increasing levels of NO formed when the substrate concentrations in the external medium were increased (Supplementary Fig. 16).

Given that the H_2_O_2_ signal downstream of the HRP-CV-containing middle layer could be effectively removed by catalase activity in the inner layer, we exploited the model prototissue vessel as a dual-input/single-output device for the chemically mediated signal processing of NO production in the central lumen. We investigated four different input combinations using a fixed GOx-CV/HRP-CV/CAT-CV outer/middle/inner layer spatial sequence. The absence of at least one of the substrates resulted in a failure to generate NO (Fig. 3h). Only the dual input of glucose and hydroxyurea gave a distinct output of NO in the internal channel, corresponding to an AND logic gate operation (Supplementary Fig. 17). In contrast, dual inputs in the absence of catalase-decorated CVs in the inner module produced a mixture of NO and H_2_O_2_ in the lumen, while only a H_2_O_2_ output was observed when the HRP- and CAT-decorated CVs were absent from the middle and inner layers, respectively (Supplementary Fig. 18). No products were detected for glucose/hydroxyurea dual inputs in the absence of GOx-CVs or HRP-CVs in the outer and middle layers, respectively, or when GOx and HRP, or GOx and catalase, were absent (Supplementary Fig. 18).

**Fig. 4:**
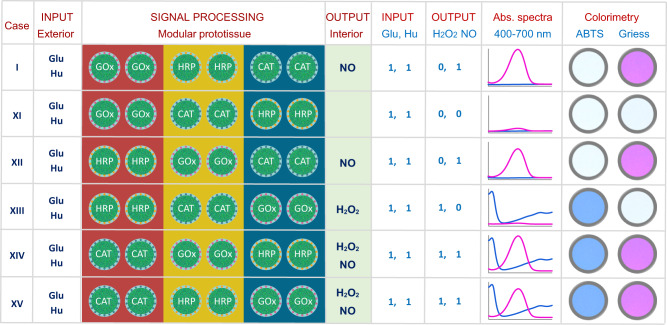
Spatial organization and signal processing in dual input model prototissues. Six different three-layer sequence arrangements of enzyme-CV hydrogel modules (case I (standard configuration; alternative configurations (cases XI-XV)) operating under dual substrate inputs are shown. Outputs in the interior lumen include NO (I and XII), H_2_O_2_ (XIII), or mixtures of H_2_O_2_ and NO (XIV and XV), depending on how the spatial sequence influences enzyme-mediated processing within the tubular micro-reactor. No outputs in the lumen are recorded for case XI. Labels and column representations are as shown in Fig. 3h. Reaction conditions: Glu (50 mM); Hu (6 mM); GOx (GOx-CV layer, 0.2 mg mL^−1^); HRP (HRP-CV layer, 0.2 mg mL^−1^); CAT (CAT-CV layer, 0.1 mg mL^−1^).

We investigated whether changes in the spatial sequence of the enzyme-CV-containing hydrogel modules gave rise to different signal outputs in the prototissue lumen under dual input conditions (Fig. 4). Switching the outer GOx-CV and middle HRP-CV modules while retaining the CAT-CV inner layer also gave a distinct NO output in the central channel, indicating upstream transfer of H_2_O_2_ into the outer HRP-CV layer and complete H_2_O_2_ decomposition in the inner module. Under these conditions, isotropic diffusion of NO from the outer layer ultimately gave rise to the detection of NO in the interior lumen. In contrast, switching the HRP-CV and CAT-CV modules whilst retaining the outer GOx-CV layer produced no NO or H_2_O_2_ outputs in the lumen due to complete depletion of the H_2_O_2_ signal during diffusion through the middle CAT-CV layer. Spatial sequences in which the CAT-CV module was positioned as the outer layer produced a mixture of NO and H_2_O_2_ in the interior channel, while H_2_O_2_ alone was detected for an outer/middle/inner layer sequence corresponding to HRP-CV/CAT-CV/GOx-CV, respectively.

### Anti-coagulation activity in NO-producing model prototissues

**Fig. 5:**
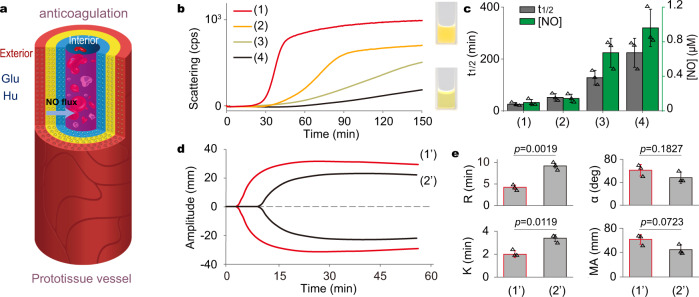
Anti-coagulation activity in NO-producing model prototissues. **a** Graphic of the model prototissue vessel used for the NO flux-mediated anticoagulation of plasma or blood samples placed in the central lumen. Populations of GOx-CVs, HRP-CVs or CAT-CVs are immobilized in the outer, middle or inner hydrogel modules, respectively. Glucose (Glu) and hydroxyurea (Hu) are added to the exterior side of the vessel. **b** Representative plots showing real-time light scattering (counts per second) of blood plasma anticoagulation in the central lumen of the prototissue-like vessel after exposure to a NO flux for different time intervals; (1) no exposure (control, red line), (2, orange) 50 min, (3, Khaki) 100 min and (4, black) 150 min, showing increasing levels of anti-coagulation (reduced light scattering). Coagulation is activated upon addition of CaCl_2_ (0.02 mol L^−1^) after exposure to NO in lumen. Inset; photographs of the plasma in cuvettes for the control sample (1) and after 150 min in the model prototissue (4). **c** Plots of half-life for plasma coagulation (*t*_1/2_) and final NO concentrations in the lumen for samples (1–4). *t*_1/2_ was obtained through fitting of a single exponential equation. **d** Four-channel recording showing TEG profiles for whole blood placed in the central lumen of the prototissue-like vessel and after exposure to a NO flux for different time intervals followed by addition of Ca^2+^ ions; (1’) (red line, control sample without NO) and (2’) (black line, 150 min). **e** Quantitative histogram analysis of the TEG profiles shown in (**d**); reaction time for fibrin formation (*R*), clot formation time (*K*), rate of clot formation (α angle), and mechanical strength of the clot (maximum amplitude, MA). Prototissue vessel composition: Glu (50 mM); Hu (6 mM); GOx (GOx-CV layer, 0.2 mg mL^−1^); HRP (HRP-CV layer, 0.2 mg mL^−1^); CAT (CAT-CV layer, 0.1 mg mL^−1^). Data are presented as mean ± s.d., two-sided Student’s t-test (*n* = 3 independent experiments).

Given the known anti-coagulant properties of NO at low dose concentrations (Supplementary Fig. 19)^52,53^, we exploited the model prototissues as tubular micro-reactors capable of inhibiting fibrin formation and thrombin-mediated platelet activation. To demonstrate bioactivity, we loaded solutions of fresh rabbit plasma or whole blood into the central cavity of the model prototissue and then added glucose and hydroxyurea to the external environment to generate a sustainable flux of NO into the lumen (Fig. 5a). Aliquots of plasma were removed after different time intervals up to 150 min, and NO-induced anti-coagulation monitored by addition of an activator (Ca^2+^ ions) to initiate a potential clotting cascade, which was monitored by light scattering. Compared to control plasma solutions that were not exposed to NO and which coagulated after addition of aqueous CaCl_2_, generation of NO within the model prototissue progressively increased the half-life for plasma coagulation (*t*_1/2_). For example, operating the prototissue-like vessel for 150 min produced NO concentrations in the lumen of *ca*. 1 μM. This resulted in a decrease in the scattering profile from approximately 116 (control without NO) to 4 cps/min, 30 min after addition of Ca^2+^ ions (Fig. 5b). Under these conditions, *t*_1/2_ was extended from 32 (control) to 280 min (Fig. 5c), indicating that the model prototissue vessel was capable of effective NO-mediated anti-coagulation activity. No anticoagulation was observed in control experiments undertaken in the absence of the enzyme substrates (Supplementary Fig. 20).

In related studies, we used thromboelastography (TEG) to determine the inhibition kinetics of clot formation in whole blood samples placed within the internal channel of the model prototissue. Fresh citrated blood was placed in the vessel lumen and exposed to the NO flux for 150 min, followed by addition of Ca^2+^ ions and transfer of the blood to the TEG hemostatic assay (Fig. 5d). The viscoelastic properties of whole blood clot formation under low shear stress were quantified by measurement of the reaction time required for initial fibrin formation (*R*), the time required to achieve a certain level of clot strength (*K* time; at an amplitude of 20 mm), the rate of clot formation (angle α), and the ultimate mechanical strength of the clot formed (maximum amplitude, *MA*). Values for *R* and *K* increased in the NO– generating prototissue-like vessel and were accompanied by decreases in α and MA values (Fig. 5e), indicating efficient anticoagulation activity. In particular, the extension in *K* from 2.0 min (control) to 3.4 min in the vessel and corresponding decrease in α from 60 to 48° confirmed the strong NO-mediated inhibition of fibrin-mediated blood clotting.

## Discussion

In this Article, the immobilization and spatial distribution of protocell colonies within concentrically arranged tubular hydrogel modules is used to construct a functional synthetic prototissue model capable of endogenous signal processing and generation of nitric oxide under reaction-diffusion conditions. The model prototissue is based on a type of protocell construct involving the surface decoration of phospholipid-enveloped PDDA/DNA coacervate droplets with lipophilically modified enzymes that collectively process a dual glucose/hydroxyurea input, which under certain conditions results in a distinct NO output in the central channel of the prototissue-like vessel. Transposing or removing any of the hydrogel modules results in different outputs, indicating that the functionality of the model prototissue depends on the spatial organization of the processing domains when operating under enzyme substrate gradients. As a specific outcome of prototissue vessel functionality, we show that a sustainable flux of bioactive NO leads to the inhibition of blood coagulation in samples located in the internal lumen of the device, demonstrating proof-of-concept use as an on-site anticoagulation device, provided that the residence times of samples within the inner channel are commensurate with the rate of blood clotting. Moreover, as the mediation of physiological processes by NO is dose-dependent and toxic at high gas concentrations, it should be possible to use the model prototissue vessel to optimize the flux of NO to address the particular requirements of specific biomedical antithrombotic applications. More broadly, our results highlight opportunities to develop spatially segregated synthetic prototissue models from modules comprising immobilized assemblages of artificial protocells and provide a step towards the organization of biochemical processes in integrated micro-compartmentalized media, micro-reactors, and soft functional materials.

In general, decoration of the phospholipid-enveloped PDDA/DNA coacervate droplets with lipophilically modified enzymes provides a strategy for locally concentrating biomolecules across the surface of a protocell membrane to generate functional micro-compartmentalized objects. By attaching the enzymes to the coacervate surface rather than employing a homogeneous dispersion of biomolecules within a coacervate-free hydrogel, soft materials with increased levels of internal organization can be generated. For example, the surface-adsorbed enzymes can be coupled with other chemistries localized within the interior of the coacervate droplets to produce embedded signalling and network systems that are spatially distributed as reaction hot-spots throughout the hydrogel.

Taken together, our results illustrate an approach to the construction of model prototissues based on the concept of protocell/hydrogel modularization. Modularity is a major factor in the adaptability and resilience of living systems and is potentially a key requirement for implementing prototissue designs across diverse biomimetic applications. For example, given that enzyme processing in the tubular prototissue-like vessels can be regulated by employing different spatial sequences of the enzyme-CV-containing hydrogel modules, it seems feasible that the prototissue models could be individually designed for integration into biomedical and pharmaceutical applications involving glucose-mediated metabolic pathways. Moreover, designing synthetic prototissues with bespoke shape, size, spatial configuration and micro-anatomy could provide a route to artificial constructs that complement the use of organoids as therapeutic models in regenerative medicine^54^, or serve as micro-reactor-based implants for diagnostic, therapeutic, and theranostic applications.

## Methods

### Materials

The following chemicals were used as received: poly(diallyldimethylammonium chloride) solution (PDDA, average Mw 200–350 kDa, 20 wt% in H_2_O, Sigma); double-stranded deoxyribonucleic acid (DNA, low molecular weight from salmon sperm, Sigma); fluorescein-5-isothiocyanate (FITC, Thermo Fisher), Hoechst 33258 (*ds*DNA binding fluorescent dyes, Thermo Fisher), 2,2’-azino-bis(3-ethylbenzothiazoline-6-sulfonic acid) (ABTS, Roche), sulforhodamine B (dye content 75%, Sigma), Congo red (dye content, ≥85%, Sigma), direct yellow 27 (Sigma), Brilliant blue R (Dye content ~50%, technical grade, Sigma), Griess reagent (colorimetric probe for measuring nitrite content (NO production), modified with 0.2% naphthyl ethylenediamine dihydrochloride and 2% sulphanilamide in 5% phosphoric acid), 1,1′-dioctadecyl-3,3,3′,3′-tetramethylindocarbocyanine perchlorate (DiI, lipid bilayer membrane fluorescence probe, Thermo Fisher); 1,2-dioleoyl-sn-glycero-3-phosphocholine (DOPC, Avanti Polar Lipids), agarose (low gelling (congealing) temperature, 26–30 °C, Sigma), hydroxyurea (98%, powder, Sigma), glucose (D-(+)-glucose, anhydrous, ≥99.5%, BioUltra), glucose oxidase (GOx, from *Aspergillus niger*, lyophilized, powder, ~200 U mg^−1^, Sigma), horseradish peroxidase (HRP, lyophilized, powder, beige, ~150 U mg^−1^, Sigma), catalase (CAT, lyophilized powder, 2000–5000 U mg^−1^, Sigma), palmitic acid N-hydroxysuccinimide ester (PA-NHS, ≥ 98%, Sigma). New Zealand white rabbits were purchased from Hunan Taiping Biological Technology Limited Company. Milli-Q-purified water (18.2 MΩ cm) was used for all the experiments.

### Characterization

UV-vis adsorption and transmittance measurements were carried out using a PerkinElmer Lambda 25 spectrophotometer. Fluorescence spectra and time scanning were conducted in a steady state spectrofluorometer (Horiba, FluoroMax). Zeta potential and dynamic light scattering (DLS) measurements were performed using a Malvern Zetasizer Nano-ZS instrument equipped with a 633 nm laser. Optical and fluorescence measurements were performed on a Nikon TE-2000U inverted microscope. The fluorescence images were captured on a C9100-13 EMCCD camera (Hamamatsu) or SD1600AC CDD camera (iMG). The fluorescence imaging data were analyzed using ImageJ software. Dark-field imaging was conducted in an Olympus Model BX51 upright microscope. Laser confocal fluorescence microscopy measurements were performed using a Leica SP5-II AOBS confocal laser scanning microscope attached to a Leica DM I6000 inverted epifluorescence microscope. Three-dimensional reconstructions from Z stacks were performed using ImageJ (plugin 3D viewer). Scanning electron microscopy (SEM) images were carried out on a JEOL SEM 5600 field emission scanning electron microscope with 20 keV acceleration voltage. Rheology measurements were performed using a Malvern Kinexus Pro+ geometry parallel plate rheometer. The test geometry was a 40 mm diameter plate.

### Preparation of phospholipid-enveloped coacervate vesicles

Membrane-less PDDA/DNA coacervate micro-droplet dispersions were prepared with different zeta potentials by mixing aqueous solutions of PDDA (average Mw 200–350 kDa, 0.8% wt in water, 50 mM, 8 mg mL^−1^, pH = 8.0) and *ds*DNA (low Mw (salmon sperm), 5 mg mL^−1^, pH = 8.0) at different weight ratios (PDDA: DNA = 0.4-0.8, w/w). Mixing of 1.0 mL of 8.0 mg mL^−1^ PDDA (pH = 8.0) and 2.6 mL of 5.0 mg mL^−1^ DNA (pH = 8.0) resulted in the formation of positively charged coacervate microdroplets (7.2 mg mL^−1^) that were used for most of the experiments.

DOPC-encapsulated coacervate vesicles (DOPC-CVs) were prepared as follows. 1 mL of a suspension of positively charged PDDA/DNA coacervate microdroplets (PDDA: DNA = 0.4-0.8, w/w, 7.2 mg mL^−1^, pH = 8.0) was added to 36 µL of 20 mg mL^−1^ DOPC ethanol solution, achieving a final weight ratio of 10% (DOPC: coacervate, w/w). After aging for more than 2 h at room temperature, a suspension of membranized coacervate vesicles (8.0 mg mL^−1^) was obtained. The number density of the coacervate vesicles in suspension was determined using optical microscopy^50^.

### Hydrophobic modification of enzymes

Hydrophobic modification of enzymes with palmitic acid ester was performed as follows^55^. 0.4 mL of palmitic acid N-hydroxysuccinimide ester (PA-NHS) solution in dry dimethyl sulfoxide (four portions of 0.1 mL every 2 h) was added to 6 mL of enzyme solution (4 mg mL^−1^ GOx, HRP or CAT) in 100 mM phosphate buffer in the presence of 1% (w/w) deoxycholate (pH 8.8). The molar ratio of enzymes to the ester in the reaction mixture was 1:40. The reaction mixture was kept for 8 h in a thermostat at 30 °C while stirring. The mixture was then filtered through a 0.45 mm filter, and dialyzed with 50 mM phosphate buffer (pH 8.0) for 3 times. After dialysis, the solution containing the hydrophobized enzyme (PA-GOx, PA-HRP or PA-CAT) was filtered through a 0.2 µm filter, and then lyophilized and stored in powder form. The products were characterized by matrix-assisted laser desorption/ionization time of flight mass spectrometry (MALDI-TOF MS), which was performed on a 4700 Proteomics analyzer (Applied Biosystems) using 2,5-dihydroxybenzoic acid as matrix substance. The sample aqueous solution concentration was 5.0 mg mL^−1^.

FITC labelling of the PA-enzymes was conducted during the PA-NHS conjugation step. 1 mg mL^−1^ of FITC in dimethyl sulfoxide together with PA-NHS was added to the enzyme solution to give a final concentration of 100 µg FITC/1 mg enzyme. The sample tube was wrapped in foil and mixed at room temperature for 2 h. Unreacted FITC was removed by ultrafiltration using Millipore Amicon-Ultra-15 tubes (MWCO = 10 kD) to give the FITC-labelled PA-enzymes which were collected and stored in 50 mM PBS buffer at −20 °C for further use. FITC labelling of the native enzymes was also undertaken but without use of PA-NHS.

### Decoration of DOPC-CVs with hydrophobically modified enzymes

Attachment of the palmitic acid (hexadecanoic acid)-modified enzymes to the outer membrane surface of the DOPC-CVs was performed as follows. 1 mL suspension of DOPC-CVs (8.0 mg mL^−1^, pH = 8.0) was separately added to 5 mg mL^−1^ PA-enzyme solution (100 µL for PA-GOx, 100 µL for PA-HRP, and 50 µL for PA-CAT). After incubation for 60 min and subsequent centrifugation for 5 min at 20 × *g*, enzyme-decorated DOPC-CVs (PA-GOx-CVs, PA-HRP-CVs, and PA-CAT-CVs) were obtained. Due to incomplete adsorption and a limit on the loading efficiency, the final enzyme loading values obtained from adsorption experiments were around 0.4 mg mL^−1^ GOx for PA-GOx-CVs, 0.4 mg mL^−1^ HRP for PA-HRP-CVs, and 0.2 mg mL^−1^ CAT for PA-CAT-CVs.

### Preparation of protocell-based hydrogel modules

Hydrogel modules were prepared by immobilization of enzyme-decorated coacervate vesicles in agarose. Agarose powder (low gelling temperature) was poured into a conical flask along with Milli-Q water and heated in a microwave oven for 1–3 min until the solution was gently boiling and the agarose was completely dissolved to produce a 2 wt% solution. The agarose solution was then cooled to *ca*. 40 °C in a water bath. 1 mL of the warm agarose solution was then immediately added to 1 mL of an aqueous suspension of enzyme-decorated DOPC-CVs (vesicle concentration, 8.0 mg mL^−1^; enzyme concentrations 0.4 mg mL^−1^ (GOx), 0.4 mg mL^−1^ (HRP), 0.2 mg mL^−1^ (CAT)) in a glass tube (inner diameter 15 mm) with a removable plastic stopper at one end, well-mixed, and then left at 4 °C in a fridge for approximately 30 min during which hydrogelation (final concentration, 1 wt% agarose) occurred along with entrapment of the coacervate vesicles. The resulting hydrogel was obtained from the glass tube as a self-supporting material by removing the plastic stopper and gently displacing the hydrogel using a glass rod.

Agarose hydrogels were prepared using concentrations of 0.5 to 4 wt% (usually 1 wt%), depending on individual experiments. Hydrogel modules were also prepared with a final coacervate vesicle concentration of 4.0 mg mL^−1^ and final enzyme concentrations of 0.2 mg mL^−1^ (GOx), 0.2 mg mL^−1^ (HRP), and 0.1 mg mL^−1^ (CAT). In general, the enzyme loading was adjusted by changing the volume of the protocell suspension added to the hydrogel mixtures. For example, adding 0.25 mL of the warm agarose solution to a 0.75 mL of an aqueous suspension of HRP-CVs (coacervate vesicles, 8.0 mg mL^−1^; HRP, 0.4 mg mL^−1^) resulted in HRP-CVs hydrogel modules with a HRP concentration of 0.3 mg mL^−1^. Similarly, 0.95 mL of the warm agarose solution was added to a 0.05 mL of an aqueous suspension of CAT-CVs protocells (coacervate vesicles, 8.0 mg mL^−1^; CAT, 0.2 mg mL^−1^), resulting in the formation of CAT-CVs hydrogel modules with a CAT concentration of 0.01 mg mL^−1^.

Enzymatic activities of GOx and HRP in the hydrogel modules were evaluated using a H_2_O_2_-ABTS colorimetric assay. The enzyme activity of CAT in the hydrogel module was determined through changes in adsorption at 240 nm (H_2_O_2_).

### Design and construction of a tubular prototissue vessel

A model prototissue vessel was assembled from three concentric hydrogel modules containing GOx-CVs, HRP-CVs, or CAT-CVs arranged respectively from the exterior to interior of the vessel using a gel perfusion method involving four steps: (1) A 15 mm-diameter glass tube glass tube (length, 100 mm) was sealed at one end by a plastic stopper and stood upright in an agarose hydrogel matrix. (2) A 12 mm-diameter glass rod (length, 90 mm) was placed in the centre of the glass tube and 10 mL of a hot (40 °C) aqueous agarose suspension containing GOx-CVs was added to fill the empty space between the glass tube and inserted glass rod, and then cooled to 4 °C for 30 min in a fridge to induce hydrogelation. The 12 mm-diameter glass rod was then carefully removed to produce a tubular outer layer comprising a hydrogel/GOx-CV module. (3) A 9 mm-diameter rod (length, 90 mm) was then placed into the centre of the glass tube, and 7 mL of a hot (40 °C) aqueous agarose suspension containing HRP-CVs added to fill the empty space between the outer GOx-CV-containing hydrogel layer and inserted glass rod, and then cooled to 4 °C for 30 min in a fridge to induce hydrogelation. The 9 mm-diameter glass rod was then carefully removed to produce a tubular middle layer consisting of a hydrogel/HRP-CV module. (4) A 6 mm-diameter glass rod (length, 90 mm) was placed in the centre of the glass tube and 2 mL of a hot (40 °C) aqueous agarose suspension containing CAT-CVs added to fill the empty space between the middle HRP-CV -containing hydrogel layer and inserted glass rod, and then cooled to 4 °C for 30 min in a fridge to induce hydrogelation. The 6 mm-diameter glass rod was then carefully removed to produce a tubular inner layer consisting of a hydrogel/CAT-CV module. Finally, the resulting tubular three-layer prototissue vessel was obtained from the glass tube as a self-supporting material by removing the plastic stopper and gently removing the 15 mm-diameter glass tube. Samples were stored in the fridge prior to use. The prototissue vessels were stained by adding Congo red (0.5 mM), Direct yellow (0.5 mM) and Brilliant blue (0.5 mM) to the outer, middle and inner layers during the assembly process.

### Prototissue model-mediated processing of nitric oxide generation

A three-layer tubular prototissue vessel (length, 50 mm) comprising outer, middle and inner hydrogel layers loaded with GOx-CVs (0.2 mg mL^−1^), HRP-CVs (0.2 mg mL^−1^) or CAT-CVs (0.1 mg mL^−1^), respectively was used for the controlled production of NO in the internal lumen. The prototissue vessel was sealed at the bottom end with enzyme-free agarose hydrogel and then stood upright in a 20 mL beaker. DPBS buffer (Dulbecco’s phosphate-buffered saline buffer) containing 50 mM glucose and 6 mM hydroxyurea was added as an external solution. The volume added was such that the open end of the vertical prototissue vessel was not immersed, preventing direct access of the enzyme substrates to the central lumen. The central channel of the prototissue vessel was then filled with DPBS buffer to generate a substrate diffusion gradient across the tubular micro-reactor.

After reaction at different time intervals, the H_2_O_2_ and NO produced in the different hydrogel layers of the prototissue vessel were recorded using H_2_O_2_ and NO microelectrode sensors (World Precision Instruments). H_2_O_2_ and NO concentrations in the central lumen of the prototissue model were determined by real-time monitoring of the reaction products in the internal channel using the open end of the vertically positioned tube to collect the samples. H_2_O_2_-ABTS and Griess reagent colorimetric assays were used to determine the H_2_O_2_ and NO concentrations, respectively.

### In situ microelectrode-based monitoring of H_2_O_2_ and NO production

Real-time monitoring of H_2_O_2_ and NO within the different layers of the prototissue vessel were recorded using microelectrode sensors (electrophysiology tissue microelectrodes, World Precision Instruments). H_2_O_2_ measurements were performed using a Four-Channel Free Radical Analyzer (WPI, TBR 4100) equipped with a peroxide hydrogen sensor (ISO-HPO-100). The microelectrode has a limit of detection (LOD) of 1 nM–1 mM, response time of < 5 s (90%), drift of <1.0 pA/min, and sensitivity of 1 pA/nM. The H_2_O_2_-reactive part of the needle electrode had a proximal length of 1–5 mm with a diameter of 100 µm. The electrode was equilibrated and polarized according to recommendations from the manufacturing company (WPI), and calibrated using 3%(w/w) H_2_O_2_ solution in 100 mM PBS buffer.

NO measurements were performed using a Four-Channel Free Radical Analyzer (WPI, TBR 4100) equipped with nitric oxide sensor (ISO-NOPF200). The microelectrode has a LOD of 0.2 nM, and Ag-AgCl was used as a reference electrode. The NO-reactive part of the needle had a proximal length of 1–5 mm with a diameter of 200 µm, which was coated in a hydrophobic gas permeable membrane. The instrument calibration was performed at 37 °C. The electrode was equilibrated and polarized according to recommendations from the manufacturing company (WPI). For calibration, S-nitroso-N-acetyl-d,l-penicillamine (SNAP) was used in combination with the catalyst cuprous (I) chloride (CuCl) to generate a known amount of NO in solution.

### Colorimetric assays of H_2_O_2_ and NO production in the prototissue lumen

H_2_O_2_ concentration in the lumen of the prototissue vessel was determined by using a H_2_O_2_-ABTS colorimetric assay. The solution collected from the lumen was ultra-filtrated to remove any enzymes that may have leached into the internal channel. Before H_2_O_2_ determination, NO was removed through addition of Carboxy-PTIO, a nitric oxide radical scavenger. Samples (200 µL) were then placed in the wells of a 96-well clear microplate, and HRP (final concentration 50 ng mL^−1^) and ABTS (final concentration 2 mM) then added into each well. After 20 min, the optical density (OD) was measured at λ = 418 nm and compared to a standard curve. The amount of H_2_O_2_ produced was estimated by monitoring the absorption of the ABTS diradical at λ = 418 nm (*ε*_418_ = 36,800 M^−1^ cm^−1^).

The NO concentration in the lumen was determined by the Griess reagent colorimetric assay. The Griess reagent was prepared by combining equal amounts of N-1-(naphthylethyl)ethylenediamine and sulfanilamide. Samples (100 µL) were placed in the wells of a 96-well clear microplate and Griess reagent (100 µL) added to each well. After 15 min, the optical density (OD) was measured at λ = 540 nm and compared to a standard curve.

### Anticoagulation activity in NO-producing tubular prototissue vessels

All procedures with the animal experiments in this study were performed in accordance with the guidelines of the National Institutes of Health for the Care and Use of Laboratory Animals and were approved by the Ethics Committee on Animal Care of Hunan University (No. SYXK (Xiang) 2018-0006).

Blood was collected through venipuncture in New Zealand white rabbits using a 21-gauge butterfly needle and placed in vacutainer tubes containing sodium citrate. The citrated whole blood was centrifuged at 50 × *g* for 15 min, and rabbit plasma collected from the supernatant. The plasma was added into the central channel of a tubular prototissue vessel comprising outer, middle, and inner hydrogel modules containing immobilized populations of GOx-CVs, HRP-CVs, and CAT-CVs, respectively. NO production in the central channel was initiated by the addition of glucose and hydroxyurea to the exterior side of the micro-reactor. After incubation for 90 min, 300 µL of plasma were removed from the lumen and 30 µL of 0.2 M CaCl_2_ quickly added to initiate a clotting cascade. The coagulation kinetics were monitored by light scattering using a Hitachi FL-7000 spectrofluorometer with excitation and emission wavelengths were set at 580 nm. As a control experiment, 30 µL of 0.2 M CaCl_2_ was directly added to a volume of 300 µL plasma without being exposed to NO. The initial rate of clotting was calculated from the slope of the scattering profile 30 min after the addition of the activator (Ca^2+^ ions). The half-life (*t*_1/2_) was obtained through fitting of a single exponential equation based on a one-phase exponential decay function with a time constant parameter by using Originlab. NO in the interior lumen was simultaneously determined by Griess colorimetric assay.

Anti-coagulation in citrated whole blood samples was also evaluated by thrombelastography (TEG) performed at 37 °C using a Haemostasis Analyzer (TEG-5000, Haemonetics, Braintree, MA) according to the manufacturer’s guidelines. Aliquots (1 mL) of the blood were removed from the central lumen of a NO-producing prototissue vessel after incubation for 90 min, mixed with kaolin (Cat. No. 6300, Haemonetics, Braintree, MA), and then 360 µL of the kaolin-activated plasma transferred to the pre-warmed TEG cups. 30 µL of 0.2 M CaCl_2_ was quickly added to initiate the clotting cascade^56^. As a control experiment, 30 µL of 0.2 M CaCl_2_ was directly added to 360 µL of kaolin-activated plasma without being exposed to NO. The TEG analyzer was calibrated and evaluated daily by running quality control samples before experimental sample analysis.

The following TEG parameters were determined to investigate the anticoagulation activity of NO generated from the prototissue vessels: (i) the reaction time *R*, which is the latency from start of the test to initial fibrin formation (coagulation phase I) reflecting formation of thromboplastin, thrombin, and factor Xa; (ii) *K*, the time from appearance of the first fibrin filaments to clot formation (coagulation phase II); (iii) *MA*, maximum amplitude of the clot (mm) indicating increase or decrease of fibrinogen concentration and corresponding to coagulation phase III; (iv) *S*, syneresis constant describing the entire period of fibrin coagulation; (v) *MA*, time to maximum amplitude of the clot; and (vi) *J*, the total index of coagulation calculated as *J* = 160 × tanα, where α is the angle between the horizontal line in the middle of the TEG tracing and the line tangential to the developing body of this tracing at 20 mm amplitude^57^.

### Statistical analyses

Statistical analyses were performed using Origin 6.5. Student’s t-tests were used for comparison between two groups according to data distribution. Values were normally distributed, and the variance was similar between compared groups. *P* < 0.05 was considered statistically significant. All the data were repeated at least three times on three different samples.

### Reporting summary

Further information on research design is available in the Nature Research Reporting Summary linked to this article.

## Supplementary information

Supplementary Information

Reporting Summary

## Data Availability

The authors declare that all data generated in this study are provided in the article and the Supplementary Information file, and are also available from the corresponding author upon request. Source data are provided with this paper.

## Source data

Source Data
