# Supplementary material for: Signal processing and generation of bioactive nitric oxide in a model prototissue
Source: Nat Commun. 2022 Sep 6;13:5254. doi: 10.1038/s41467-022-32941-6 (PMC9448809; doi:10.1038/s41467-022-32941-6)
Supplement: Supplementary file 1 — Supplementary Information [file 41467_2022_32941_MOESM1_ESM.pdf]

Supplementary Information

Signal processing and generation of bioactive nitric oxide in a model prototissue

Songyang Liu[1,#], Yanwen Zhang[1,#], Xiaoxiao He[1], Mei Li[2], Jin Huang[1], Xiaohai Yang[1], Kemin Wang[1], Stephen Mann[2]*, Jianbo Liu[1]*

[1] *State Key Laboratory of Chemo/Biosensing and Chemometrics, College of Chemistry and Chemical Engineering, College of Biology, Key Laboratory for Bio-Nanotechnology and Molecular Engineering of Hunan Province, Hunan University, Changsha 410082, P. R. China.*
[2] *Centre for Protolife Research, School of Chemistry and Max Planck-Bristol Centre for Minimal Biology, University of Bristol, Bristol BS8 1TS, United Kingdom.*
[3] School of Materials Science and Engineering, Shanghai Jiao Tong University, Shanghai 200240, P. R. China.
[#] *These authors contributed equally:* Songyang Liu, Yanwen Zhang.
Email: s.mann@bristol.ac.uk; liujianbo@hnu.edu.cn.

Table of Contents

Supplementary Figures..2

Supplementary References..12

Supplementary Figures

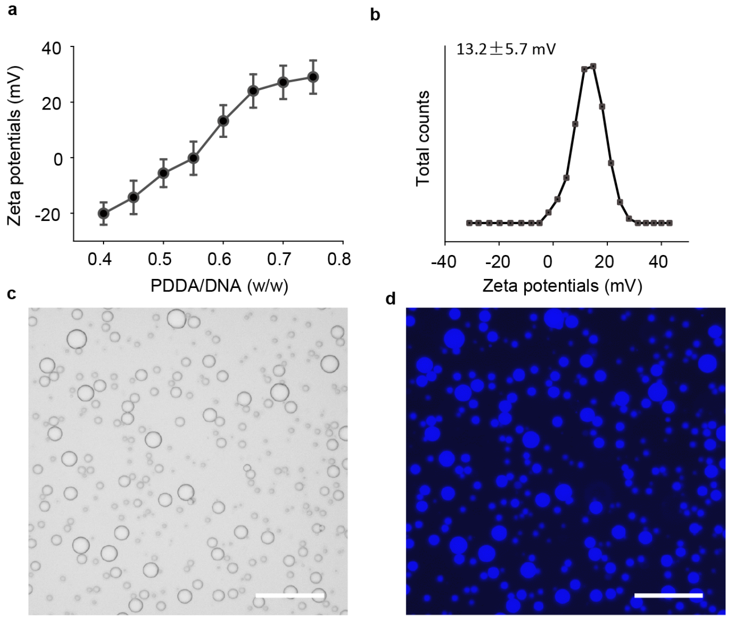

Supplementary Figure 1. Characterization of PDDA/DNA coacervate microdroplets. (**a**) Zeta potentials of PDDA/DNA coacervate microdroplets produced at different polyelectrolyte weight ratios (0.4−0.8, w/w). Data are presented as mean ± s.d. (n= 3 independent experiments). (**b**) A typical zeta potential profile of an aqueous dispersion of PDDA/DNA coacervate microdroplets (PDDA=8.0 mg mL^{-1}, DNA=5.0 mg mL^{-1}, PDDA/DNA = 0.6:1, w/w; ζ = +13.2 ± 5.7 mV) at pH=8. (**c**, **d**) Optical bright field (**c**) and corresponding blue fluorescence (**d**) images of DNA-binding Hoechst-stained PDDA/DNA coacervate microdroplets (PDDA/DNA = 0.6:1, w/w); scale bar, 10 µm. 3 times each experiment was repeated independently with similar results (**c**, **d**).

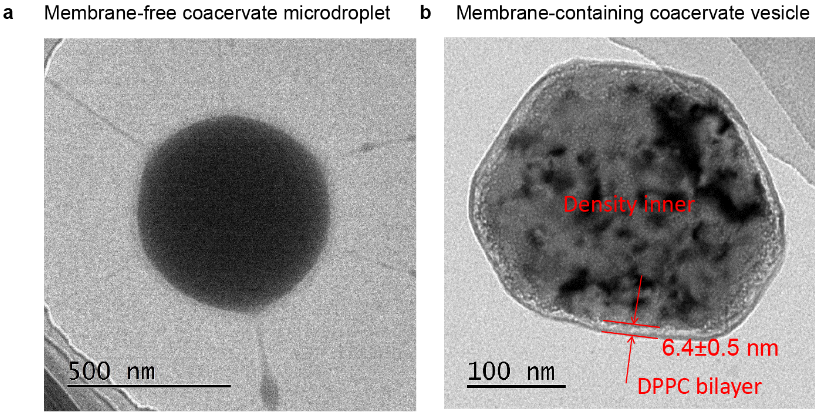

Supplementary Figure 2. TEM images of a single membrane-free coacervate microdroplet **(a)** and membrane-enveloped coacervate vesicle **(b).** The membrane-free coacervate microdroplet shows homogeneous electron density, while the membrane-enclosed coacervate vesicle shows an electron dense interior and outer membrane structure. The non-spherical shape of the coacervate vesicle is likely to be a drying artefact. The small membrane-free coacervate microdroplets are from the supernatant solution after centrufugation of an aqueous dispersion of coacervate microdrolets (PDDA=8.0 mg mL^{-1}, DNA=5.0 mg mL^{-1}. PDDA/DNA = 0.6:1, w/w) for 5 min at 1,000 rpm. 3 times each experiment was

repeated independently with similar results.

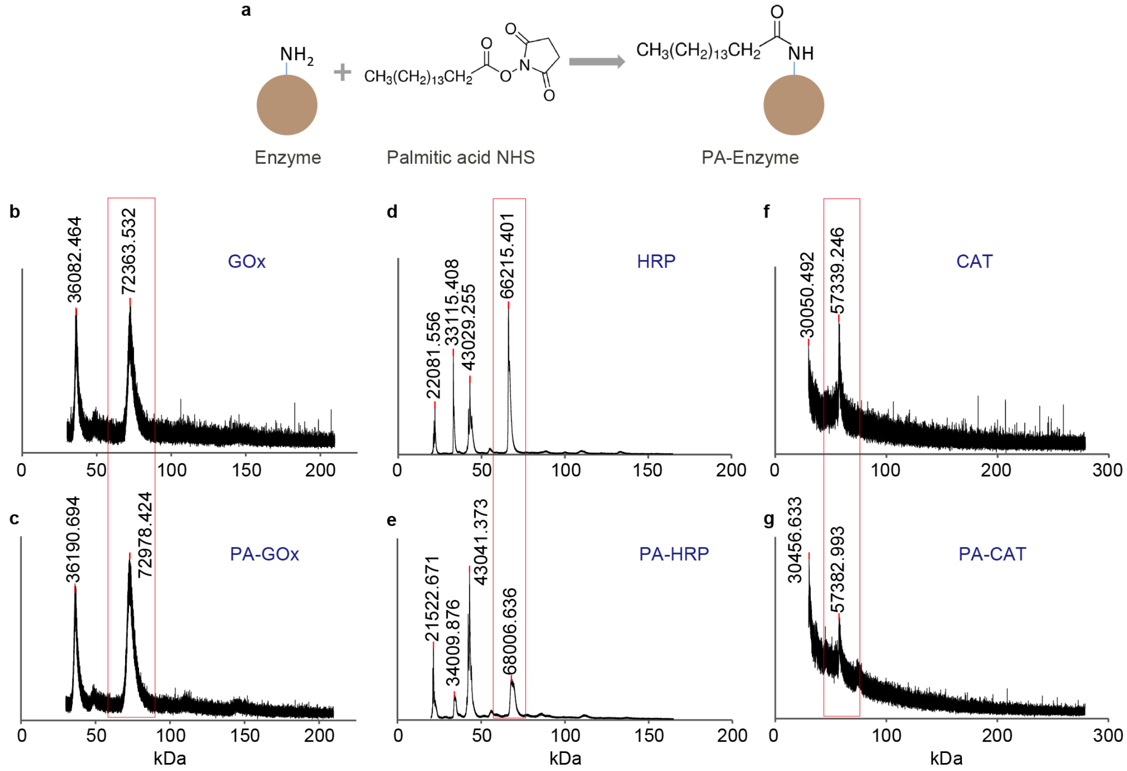

Supplementary Figure 3. Characterization of palmitic acid (PA)-modified enzymes (PA-Enzyme: PA-GOx, PA-HRP, and PA-CAT). (**a**) Reaction scheme. (**b**-**g**) MALDI-TOF mass spectra of protein enzymes and PA-Enzyme conjugates; GOx (**b**), PA-GOx (**c**), HRP (**d**), PA-HRP (**e**), CAT (**f**) and PA-CAT (**g**). The average numbers of hexadecenoic chains attached per enzyme molecule were 4.3 (PA-GOx), 6.2 (PA-HRP) and 1.6 (PA-CAT).

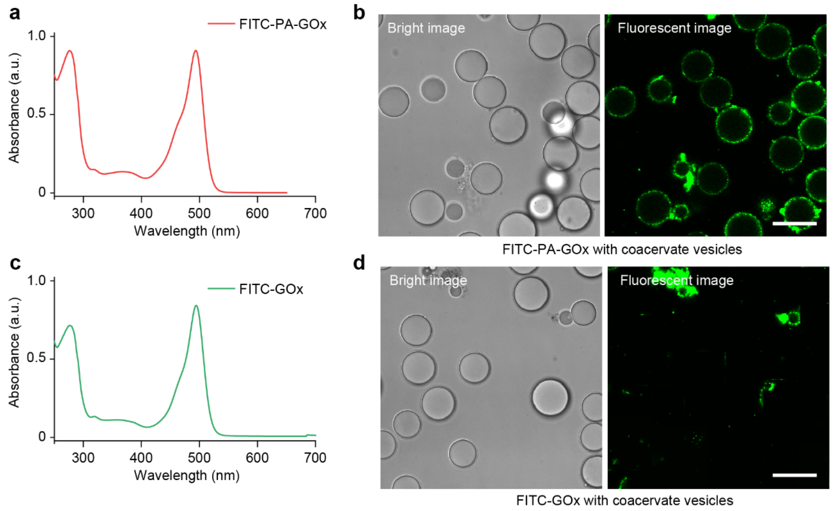

Supplementary Figure 4. (**a, c**) UV-Vis absorbance spectra of FITC-PA-GOx (red line, **a**) and FITC-GOx

(green line,**c**). The fluorescein/protein molar ratio (F/P) was determined by measuring the absorbance of the conjugate at 280 nm and 495 nm, and using Supplementary Equation S1). F/P values were 5.3 and 7.0 for FITC-PA-GOx and FITC-GOx, respectively. (**b**, **d**) Fluorescent imaging of FITC-PA-GOx (**b**), and FITC-GOx (**d**) in the presence of DOPC-CVs. A high level of FITC-PA-GOx attachment to the coacervate vesicles is observed (**b**). In contrast, FITC-GOx exhibits minimal interaction with the DOPC-CVs (**d**). FITC-PA-GOx and FITC-GOx (0.01 mg mL^{-1}) were separately incubated with an aqueous dispersion of DOPC-CVs (8.0 mg mL^{-1}) for 60 min. The coacervate vesicles were imaged directly by a Nikon fluorescence confocal microscope (Ex: blue excitation, Em: FITC channel). Scale bars (**b**, **d**), 10 µm. 3 times each experiment was repeated independently with similar results (**b**, **d**).

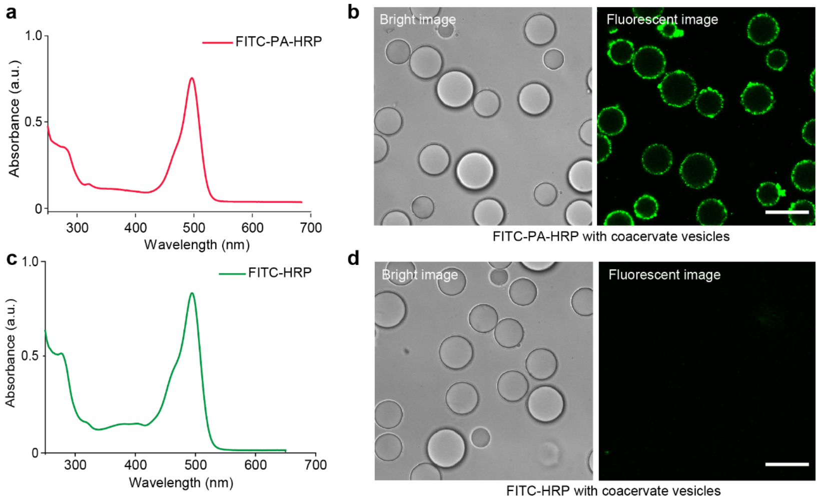

Supplementary Figure 5. (**a, c**) UV-Vis absorbance spectra of FITC-PA-HRP (red line in **a**) and FITC-HRP (green line in **c**). The fluorescein/protein molar ratio (F/P) was determined by measuring the absorbance of the conjugate at 280 nm and 495 nm. F/P values were 4.5 and 5.1 for FITC-PA-HRP and FITC-HRP, respectively. (**b**, **d**) Fluorescent imaging of FITC-PA-HRP (**b**), and FITC-HRP (**d**) in the presence of DOPC-CVs. A high level of FITC-PA-HRP attachment to the coacervate vesicles is observed (**b**). In contrast, FITC-HRP exhibits minimal interaction with the DOPC-CVs (**d**). FITC-PA-HRP and FITC-HRP (0.01 mg mL^{-1}) were separately incubated with an aqueous dispersion of DOPC-CVs (8.0 mg mL^{-1}) for 60 min. The coacervate vesicles were imaged directly by a Nikon fluorescence confocal microscope (Ex: blue excitation, Em: FITC channel). Scale bars (**b**, **d**), 10 µm. 3 times each experiment was repeated independently with similar results (**b**, **d**).

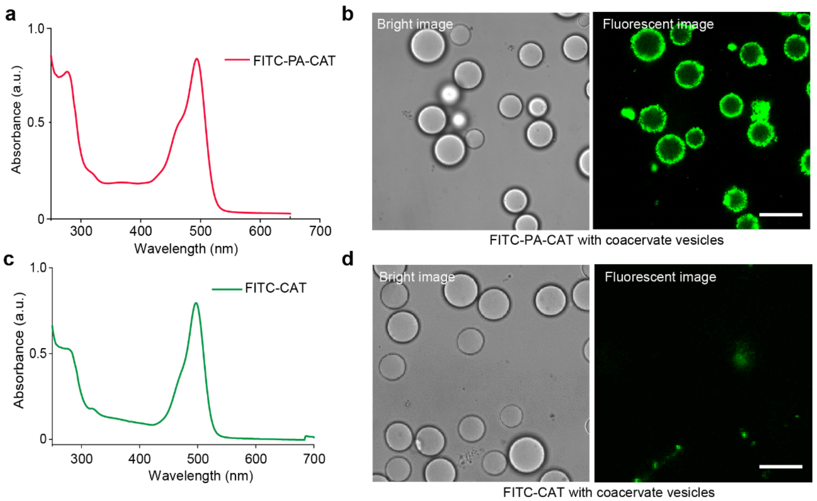

Supplementary Figure 6. (**a, c**) UV-Vis absorbance spectra of FITC-PA-CAT (red line in **a**) and FITC-CAT (green line in **c**). The fluorescein/protein molar ratio (F/P) was determined by measuring the absorbance of the conjugate at 280 nm and 495 nm. F/P values were 2.8 and 3.7 for FITC-PA-CAT and FITC-CAT, respectively. (**b**, **d**) Fluorescent imaging of FITC-PA-CAT (**b**), and FITC-CAT (**d**) in the presence of DOPC-CVs. A high level of FITC-PA-CAT attachment to the coacervate vesicles is observed (**b**). In contrast, FITC-CAT exhibits minimal interaction with the DOPC-CVs (**d**). FITC-PA-CAT and FITC-CAT (0.01 mg mL^{-1}) were separately incubated with an aqueous dispersion of DOPC-CVs (8.0 mg mL^{-1}) for 60 min. The coacervate vesicles were imaged directly by a Nikon fluorescence confocal microscope (Ex: blue excitation, Em: FITC channel). Scale bars (**b**, **d**), 10 µm. 3 times each experiment was repeated independently with similar results (**b**, **d**).

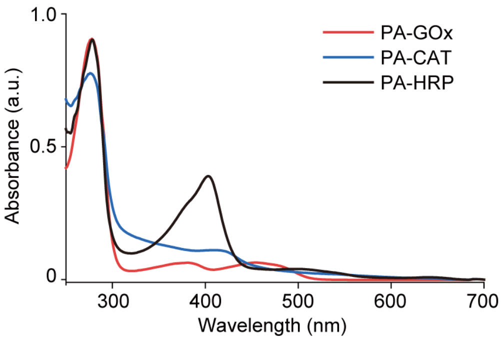

Supplementary Figure 7. UV-Vis absorbance spectra of different PA-enzymes. Characteristic absorption peaks are observed at 280 and 450 nm (PA-GOx), 280 and 403 nm (PA-HRP) and at 280 nm and 405 nm (PA-CAT). The extinction coefficients for GOx (PA-GOx) at 280 and 450 nm are 2.67×10^5 and 2.61×10^4 M^{-1} cm^{-1}, respectively.[1] The extinction coefficient for HRP (PA-HRP) at 403 nm is 1.0×10^5 M^{-1} cm^{-1}.[1] The extinction coefficient for CAT (PA-CAT) at 405 nm is 3.2×10^5 M^{-1} cm^{-1}.[2]

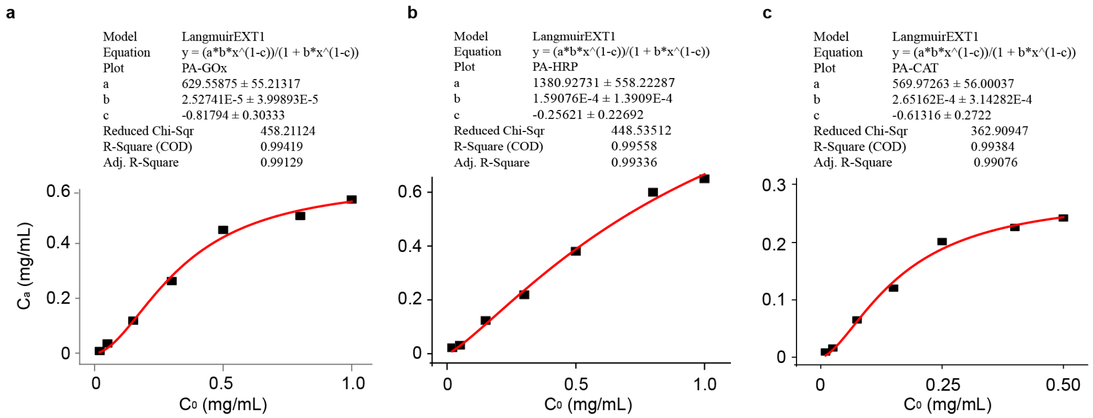

Supplementary Figure 8. Adsorption curves for PA-enzyme attachment to DOPC-CVs**.** Attachment of the hydrophobized enzymes onto the coacervate droplets is mediated by lipophilic adsorption of the PA-enzymes onto the DOPC lipid membrane of the protocells. Large volumes of the PA-enzymes (PA-GOx, PA-HRP, and PA-CAT) at different concentrations (C_0, PA-GOx, 0-1.0 mg mL^{-1}; PA-HRP, 0-1.0 mg mL^{-1}; PA-CAT, 0-0.5 mg mL^{-1}) were incubated with dispersions of DOPC-CVs (8.0 mg·mL^{-1}, pH=8.0). After incubation for 60 min, the DOPC-CVs were obtained after centrifugation for 5 min at 500 rpm. The collected enzyme-decorated DOPC-CVs were dissociated after treatment with 2.0 mol L^{-1} NaCl. The amount of enzyme adsorbed onto the coacervate vesicles (C_a) was determined from the specific absorbance peaks of the different enzymes. The extinction coefficients for GOx at 280 and 450 nm are 2.67×10^5 and 2.61×10^4 M^{-1} cm^{-1}, respectively.[1] The extinction coefficient for HRP at 403 nm is 1.0×10^5 M^{-1} cm^{-1}.[1] The extinction coefficient for CAT at 405 nm is 3.2×10^5 M^{-1} cm^{-1}.[2] The adsorption curves were fitted for each of the PA-enzymes. Non-standard Langmuir isotherms were obtained in each case. A deviation from the standard Langmuir curve is likely due to the uneven sizes of the DOPC-CVs and multi-layer adsorption of the hydrophobized enzymes. (**a**), 0.50 mg·mL^{-1} PA-GOx in a dispersion of DOPC-CVs (8.0 mg·mL^{-1}, pH=8.0) resulted in the formation of PA-GOx CVs (loading efficiency = 88%) with ~0.4 mg mL^{-1} PA-GOx immobilized on the surface of the protocell (0.4 mg mL^{-1} GOx in 4.0 mg·mL^{-1} GOx-CVs for most of the experiments). (**b**), 0.50 mg·mL^{-1} PA-HRP in a dispersion of DOPC-CVs (8.0 mg·mL^{-1}, pH=8.0) resulted in the formation of PA-HRP CVs (loading efficiency = 82%) with ~0.4 mg mL^{-1} PA-HRP immobilized on the surface of protocell (0.4 mg mL^{-1} HRP in 4.0 mg·mL^{-1} HRP-CVs for most of the experiments). (**c**), 0.25 mg·mL^{-1} PA-CAT in a dispersion of DOPC-CVs (8.0 mg·mL^{-1}, pH=8.0) resulted in the formation of PA-CAT CVs (loading efficiency = 84%) with ~0.2 mg mL^{-1} PA-CAT immobilized on the surface of protocell (0.2 mg mL^{-1} CAT in 4.0 mg·mL^{-1} CAT-CVs for most of the experiments).

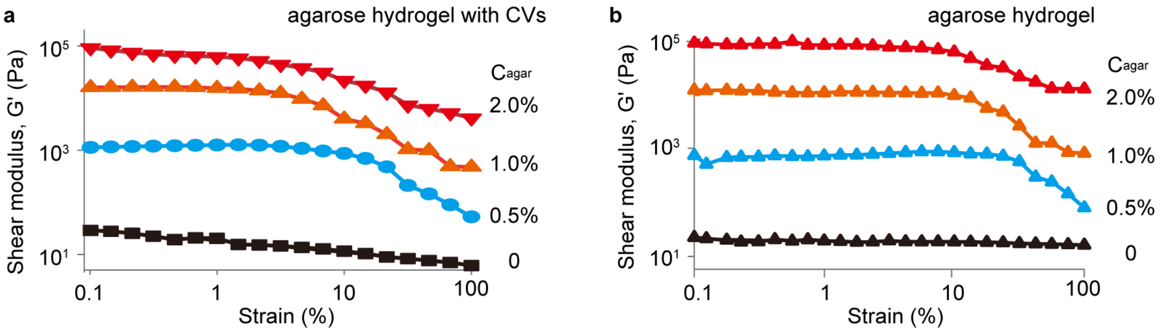

Supplementary Figure 9. Rheometry dynamic strain sweep of the storage modulus (G') for protocell-loaded hydrogels (**a**) and protocell-free hydrogels (**b**), prepared at different agarose

concentrations (red, 2.0 wt%; orange, 1.0 wt%; blue, 0.5 wt%; black 0 wt%); frequency, 1 rad·s^{-1}.

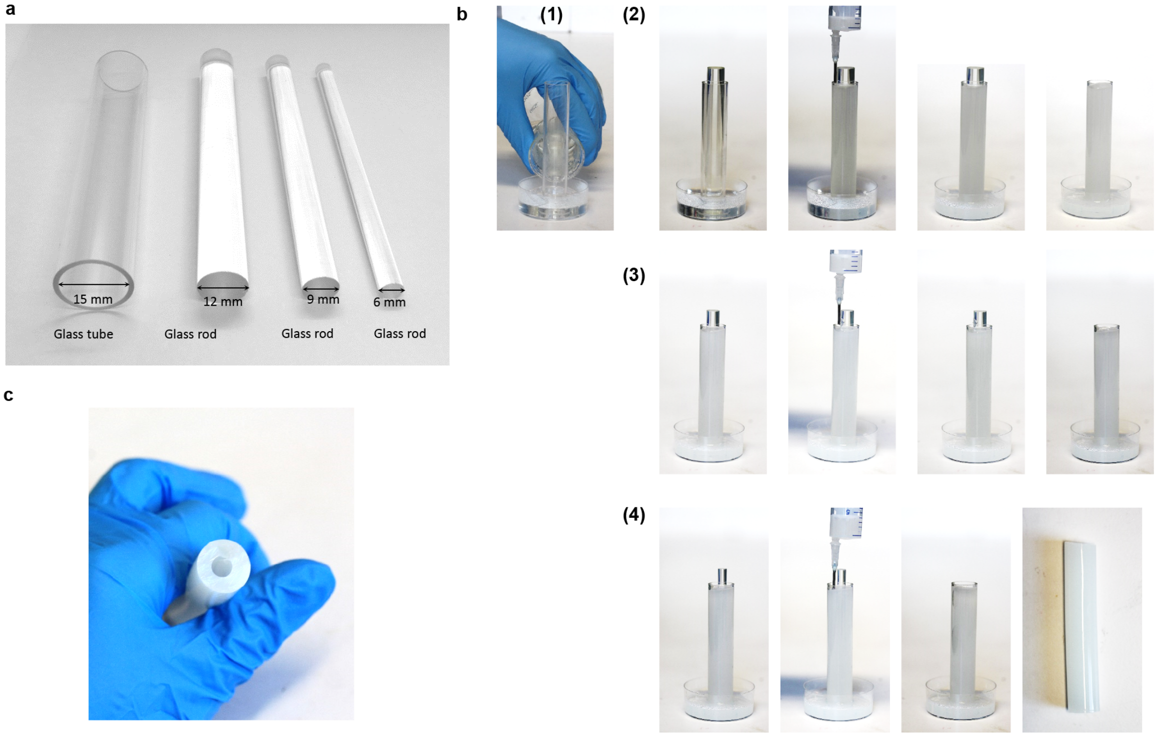

Supplementary Figure 10. Assembly of a tubular prototissue vessel from a three-layer concentric arrangement of protocell-loaded hydrogel modules. (**a**) Photograph of a 15 mm-diameter glass tube, and 12 mm-, 9 mm- and 6 mm-diameter glass rods used as physical templates for the preparation of the modular tubular prototissue vessel. Length of glass tube, 100 mm; glass rod length, 90 mm. (**b**) The prototissue vessel was assembled from three concentric hydrogel modules containing GOx-CVs, HRP-CVs, or CAT-CVs arranged respectively from the exterior to interior of the vessel using a gel perfusion method involving four steps. **Step (1)**: The glass tube is sealed at one end by a plastic stopper and stood upright in an agarose hydrogel matrix. **Step (2)**: The 12 mm-diameter glass rod is placed in the center of the glass tube and 10 mL of a hot (40 ^{o}C) aqueous agarose suspension containing PA-GOx-CVs added to fill the empty space between the glass tube and inserted glass rod, and then cooled to 4 ^{o}C for 30 min in a fridge to induce hydrogelation. The 12 mm-diameter glass rod is then carefully removed to produce a tubular outer layer comprising a hydrogel/PA-GOx-CV module. **Step (3)**: The 9 mm-diameter rod is placed into the center of the glass tube, and 7 mL of a hot (40 ^{o}C) aqueous agarose suspension containing PA-HRP-CVs is added to fill the empty space between the outer PA-GOx-CV-containing hydrogel layer and inserted glass rod, and then cooled to 4 ^{o}C for 30 min in a fridge to induce hydrogelation. The 9 mm-diameter glass rod is then carefully removed to produce a tubular middle layer consisting of a hydrogel/PA-HRP-CV module. **Step (4**: The 6 mm-diameter glass rod is placed in the center of the glass tube and 2 mL of a hot (40 ^{o}C) aqueous agarose suspension containing PA-CAT-CVs is added to fill the empty space between the middle HRP-GOx-CV -containing hydrogel layer and inserted glass rod, and then cooled to 4 ^{o}C for 30 min in a fridge to induce hydrogelation. The 6 mm-diameter glass rod is then carefully removed to produce a tubular inner layer consisting of a hydrogel/CAT-HRP-CV module. Finally, the resulting tubular three-layer prototissue vessel is obtained from the glass tube as a self-supporting material by removing the plastic stopper and gently removing the 15 mm-diameter glass tube. (**c**) Photograph showing the prototissue vessel viewed end-on. The three-layer hydrogel module arrangement has an inner channel with a diameter of 6 mm, and an outer diameter of 15 mm. Each

hydrogel layer is 1.5 mm in thickness and contains single populations of GOx, HRP or CAT-decorated DOPC-CVs. The prepared prototissue vessel was stored in a fridge.

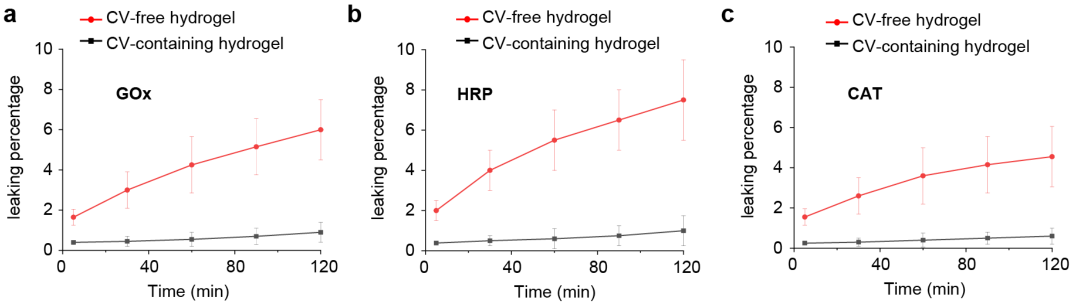

Supplementary Figure 11. Enzyme leakage experiments. Time-dependent plots of enzyme concentrations in the external solution (% leakage) for coacervate vesicle (CV)-free hydrogels containing immobilized free hydrophobized enzymes (red line) or hydrogels containing CVs with surface attached hydrophobized enzymes; (**a**) PA-GOx, (**b**) PA-HRP, (**c**) PA-CAT. The bulk hydrogels were immersed in water and the leakage determined from UV-Vis absorbance spectra of the different enzymes as shown in **Supplementary Figure 7**. Leakage of the PA-enzymes was inhibited for the CV-containing hydrogel matrix, which was attributed to attachment of the enzymes on the membrane surface of the CVs. Data are presented as mean ± s.d. (n= 3 independent experiments).

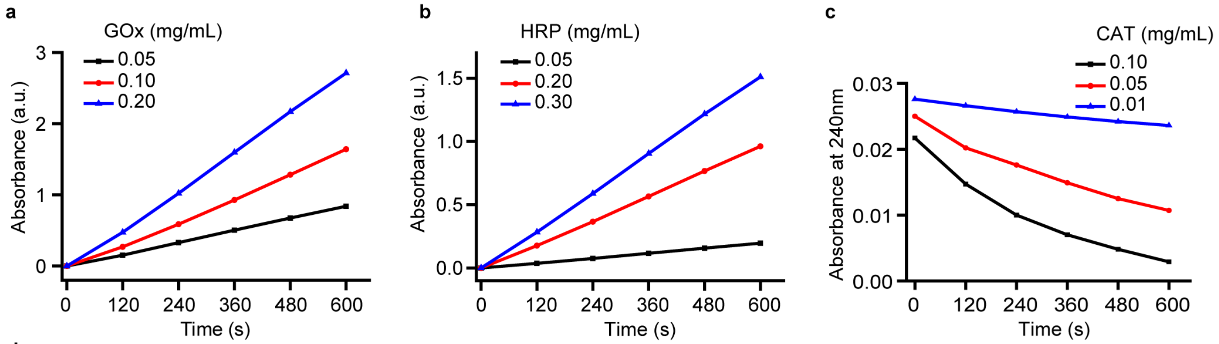

d

	GOx CVs hydrogel module			HRP CVs hydrogel module			CAT CVs hydrogel module		
Sample	(1)	(2)	(3)	(1')	(2')	(3')	(1'')	(2'')	(3'')
Enzyme (mg/mL)	0.05	0.10	0.20	0.05	0.20	0.30	0.01	0.05	0.10
delta A	0.36	0.90	1.50	0.17	0.80	1.28	0.003	0.014	0.020
Time (s)	250	250	250	250	250	250	600	600	600
k (μM/s)	0.04	0.10	0.16	0.018	0.084	0.135	0.14	0.66	0.95

Supplementary Figure 12. Activity assays for different enzyme-CV hydrogel modules. (**a**) GOx activity in GOx-CV hydrogels; GOx-CV-containing hydrogel modules (50 μL) loaded with 0.05 mg mL^{-1} GOx (black), 0.10 mg mL^{-1} GOx (red), or 0.20 mg mL^{-1} GOx (blue) were placed in 1 mL PBS buffer (150 mM, pH=7.4) along with HRP (0.02 mg mL^{-1}) and ABTS (4.0 mM). Glucose (10 mM) was then added and time-dependent changes in absorption associated with the oxidation product (ABTS-diradical) at 418 nm (ε_{418} = 36,800 M^{-1} cm^{-1}) in buffer were recorded. (**b**) HRP activity in HRP-CV hydrogels; HRP-CVs containing hydrogel modules (50 μL) loaded with 0.05 mg mL^{-1} HRP (black), 0.20 mg mL^{-1} HRP (red) or 0.30 mg mL^{-1} HRP (blue) were placed in 1 mL PBS buffer (150 mM, pH=7.4) along with ABTS (4.0 mM). H_2O_2 (10 mM) was then added and time-dependent changes in absorption associated with the oxidation product

(ABTS-diradical) at 418 nm (ε_{418} = 36,800 M^{-1} cm^{-1}) in buffer were recorded. (**c**) CAT activity in CAT-CV hydrogels; CAT-CVs containing hydrogel modules (50 µL) loaded with 0.01 mg mL^{-1} CAT (blue), 0.05 mg mL^{-1} CAT (red) or 0.10 mg mL^{-1} CAT (black) were placed in 1 mL PBS buffer (150 mM, pH=7.4). H_2O_2 (10 mM) was then added and time-dependent changes in absorption associated with the change of H_2O_2 at 240 nm (ε_{240} = 35 M^{-1} cm^{-1})[3] in buffer were recorded. (**d**) Table showing the average reaction rates (*k*) for different enzyme-CV-containing hydrogel modules under different experimental conditions, as shown in (**a-c**). The average reaction rates were estimated from H_2O_2 production or consumption in a given time interval.

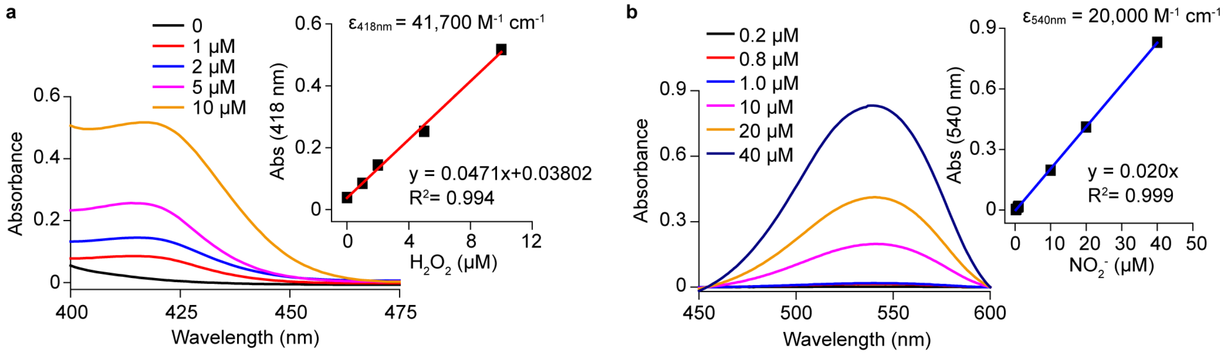

Supplementary Figure 13. Calibration curves for (a) ABTS colorimetric assay of H_2O_2 and (b) Griess colorimetric assay of NO. (a) Different concentrations of H_2O_2 (0-10 µM) were added into ABTS solution (2 mM) in the presence of HRP (100 nM) and incubated for 10 min. (b) Different concentrations of nitrite (NO_2^-) (0-40 µM) were mixed with equal volume of 1×Griess agent (40 mg/ml) and incubated for 15 min.

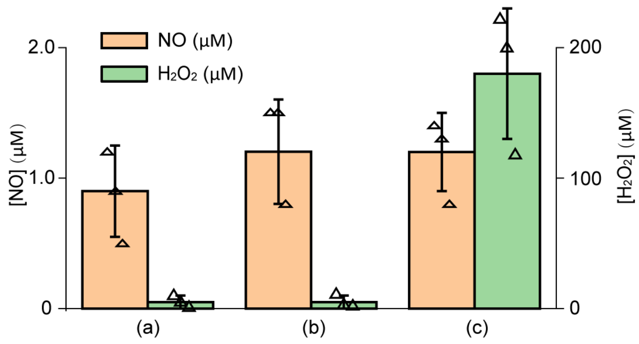

Supplementary Figure 14. Effect of changing the loading capacity in the middle (HRP-CV) and inner (CAT-CV) layers on prototissue model-mediated production of H_2O_2 and NO. Prototissue vessel composition: (**a**) 0.2 mg·mL^{-1} GOx (GOx-CV outer layer), 0.2 mg·mL^{-1} HRP (HRP-CV middle layer) and 0.1 mg·mL^{-1} CAT (CAT-CV inner layer); (**b**) 0.2 mg·mL^{-1} GOx (GOx-CV layer), 0.3 mg·mL^{-1} HRP (HRP-CV layer) and 0.1 mg·mL^{-1} CAT(CAT-CV layer); (**c**) 0.2 mg·mL^{-1} GOx (GOx-CV layer), 0.2 mg·mL^{-1} HRP (HRP-CV layer) and 0.01 mg·mL^{-1} CAT (CAT-CV layer). In each case 50 mM glucose and 6 mM hydroxyurea were added to the vessel exterior and the amounts of NO and H_2O_2 measured in the lumen were determined after 150

min. Reducing the catalase concentration in the inner layer has minimal effect on NO production but increases the amount of excess H_2O_2 produced by the micro-reactor. Data are presented as mean ± s.d. (n= 3 independent experiments).

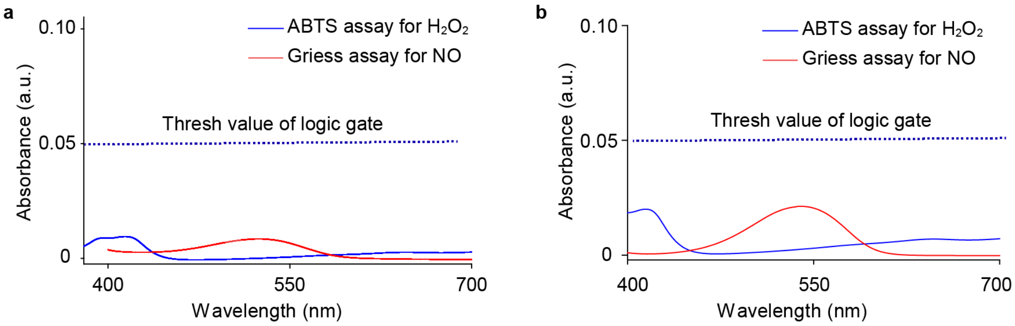

Supplementary Figure 15. Absorbance spectra recorded from HRP-mediated ABTS colorimetric assay for H_2O_2 (blue) and Griess reagent assay for NO (red). (**a**) A homogeneous mixture of GOx-CVs, HRP-CVs, and CAT-CVs in bulk solution. (**b**) Uniform distribution of the GOx-CVs, HRP-CVs, and CAT-CVs in a prototissue vessel. No apparent production of H_2O_2 and NO is observed in both cases. (GOx-CVs with 0.2 mg·mL^{-1} GOx, HRP-CVs with 0.2 mg·mL^{-1} HRP, and CAT-CVs with 0.1 mg·mL^{-1} CAT).

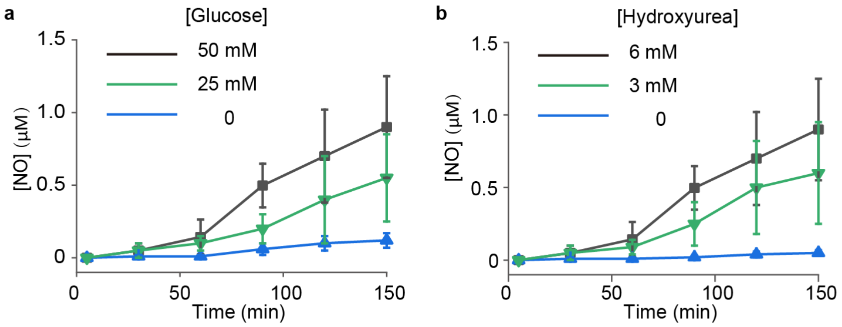

Supplementary Figure 16. Glucose-dependent (**a**) and hydroxyurea-dependent (**b**) NO generation in the interior lumen of the prototissue vessel. (**a**) addition of 0 -50 mM (black line, 50 mM; green line, 25 mM; blue line, 0 mM) glucose and 6 mM hydroxyurea to the exterior of the prototissue vessel. (**b**) addition of 50 mM glucose and 0-6 mM (black line, 6 mM; green line, 3 mM; blue line 0 mM) hydroxyurea to the exterior of the prototissue vessel. The NO concentrations were determined by NO-specific microelectrodes. Prototissue vessel composition: 0.2 mg·mL^{-1} GOx in GOx-CV layer, 0.2 mg·mL^{-1} HRP in HRP-CV layer, and 0.1 mg·mL^{-1} CAT in the CAT-CV layer. Data are presented as mean ± s.d. (n= 3 independent experiments).

a

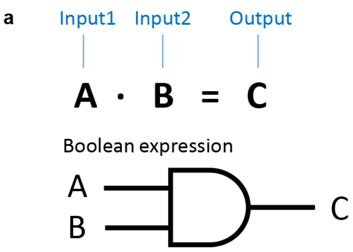

b Truth table

INPUT		OUTPUT
A	B	C
0	0	0
0	1	0
1	0	0
1	1	1

Supplementary Figure 17. (**a**) Boolean expression of an AND logic gate, and (**b**) truth table. A threshold absorption value above 0.04 at 540 nm (Greiss reagent, purple coloration with NO) was used as a verifiable (0,1) signal output.

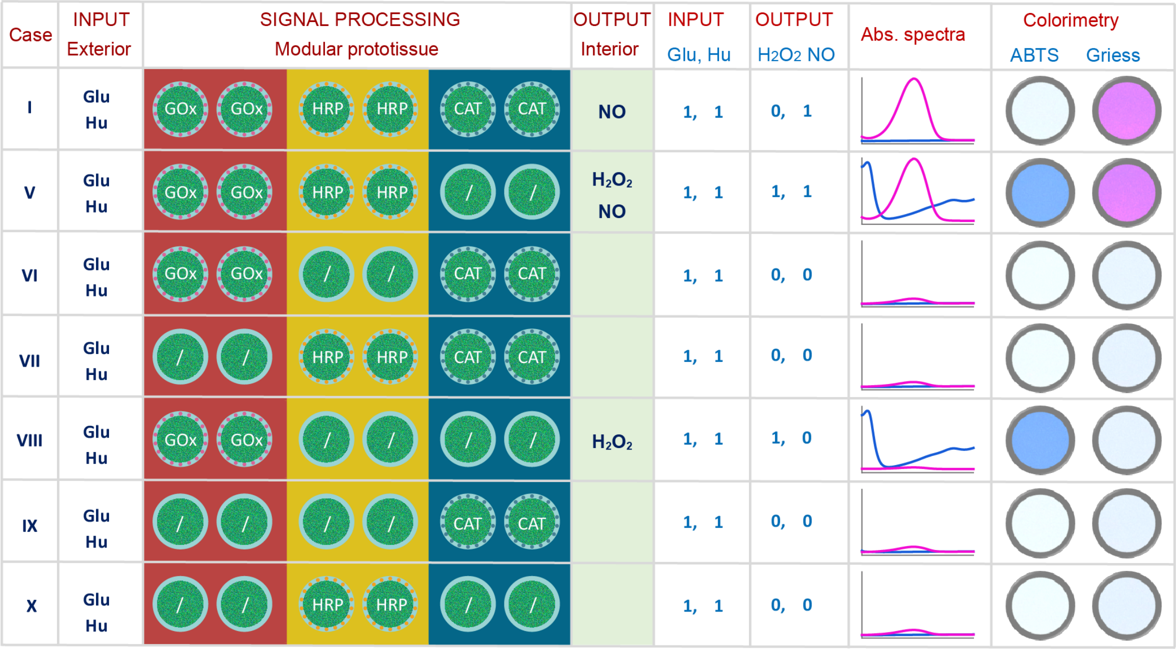

Supplementary Figure 18. Spatial organization and signal processing in dual input prototissue vessels. Seven different three-layer sequence arrangements of enzyme-CV hydrogel modules (case **I** (standard configuration; alternative configurations (cases **V**-**X**)) operating under dual substrate inputs are shown. Outputs in the interior lumen include NO (**II**), $H_2O_{2,}$ (**VIII**), or mixtures of H_2O_2 and NO (**V**), depending on how the spatial sequence influences enzyme-mediated processing within the tubular micro-reactor. No outputs in the lumen are recorded for cases **VI**, **VII**, **IX** and **X**. Labels and column representations are as shown in Figure 3h of the main manuscript. Reaction conditions: Glu (50 mM); Hu (6 mM); GOx (GOx-CV layer, 0.2 mg·mL^{-1}); HRP (HRP-CV layer, 0.2 mg·mL^{-1}); CAT (CAT-CV layer, 0.1 mg·mL^{-1}).

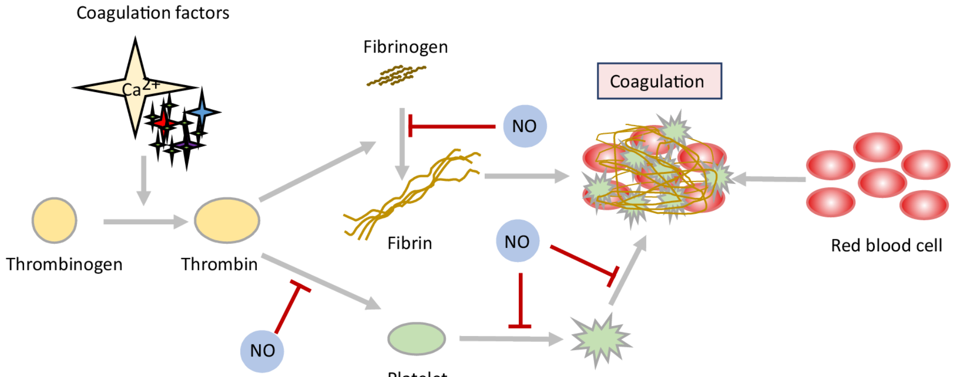

Supplementary Figure 19. Biochemical pathway for the NO-mediated anticoagulation in blood plasma. The presence of NO inhibits fibrin formation as well as thrombin-mediated platelet activation, both of which normally induce blood coagulation. Solid arrows indicate activation; bar-headed arrows indicate inhibition.[4,5]

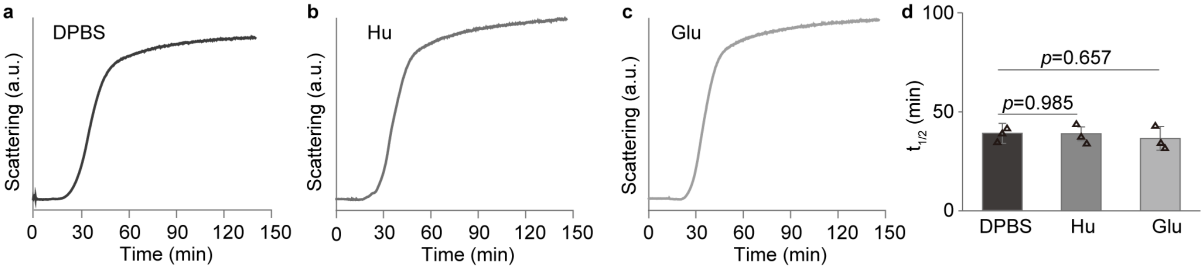

Supplementary Figure 20. Control experiments. (**a**-**c**) Real-time light scattering of blood plasma anticoagulation in the central lumen of the prototissue vessel after incubation in the lumen for 50 min. (**a**) No substrates in the exterior (DPBS buffer as control), (**b**) Hu (6 mM) in the exterior, (**c**) Glu (50 mM) in the exterior. Coagulation is activated upon addition of $CaCl_2$ (0.02 mol L^{-1}) after incubation in the lumen for 50 min. Prototissue vessel composition: GOx (GOx-CV layer, 0.2 mg·mL^{-1}); HRP (HRP-CV layer, 0.2 mg·mL^{-1}); CAT (CAT-CV layer, 0.1 mg·mL^{-1}). (**d**) Plots of half-life for plasma coagulation ($t_{1/2}$) in the lumen for samples (**a**-**c**). $t_{1/2}$ was obtained through fitting of a single exponential equation. Data are presented as mean ± s.d., two-sided Student's t-test (n= 3 independent experiments).

Supplementary References

[1] Zhang, Y., Tsitkov, S. & Hess, H. Proximity does not contribute to activity enhancement in the glucose oxidase–horseradish peroxidase cascade. *Nat. Commun.* **7**, 13982 (2016).

[2] Gębicka, L. & Gębicki, J.L. Interaction of sodium bis(2-ethylhexyljt) sulfosuccinate (AOT) with catalase and horseradish peroxidase in an aqueous solution and in the reverse micelles of AOT/n-heptane. *Biochem. Mol. Biol. Int.* **45**, 805–811 (1998).

[3] http://www.h2o2.com/technical-library/physical-chemical-properties/radiation-properties/default.aspx?pid=65&name=Ultraviolet-Absorption-Spectrum

[4] Loscalzo, J. Nitric oxide insufficiency, platelet activation, and arterial thrombosis. *Circ. Res.* **88**, 756–762 (2001).

[5] Nielsen, V.G. Nitric oxide decreases coagulation protein function in rabbits as assessed by thromboelastography. *Anesth. Analg.* **92**, 320–323 (2001).
