# Supplementary material for: Signal processing and generation of bioactive nitric oxide in a model prototissue
Source: Nat Commun. 2022 Sep 6;13:5254. doi: 10.1038/s41467-022-32941-6 (PMC9448809; doi:10.1038/s41467-022-32941-6)
Supplement: Supplementary file 2 — Reporting Summary [file 41467_2022_32941_MOESM2_ESM.pdf]

nature research

Corresponding author(s): Jianbo Liu, Stephen Mann

Last updated by author(s): Aug 22, 2022

Reporting Summary

Nature Research wishes to improve the reproducibility of the work that we publish. This form provides structure for consistency and transparency in reporting. For further information on Nature Research policies, see our Editorial Policies and the Editorial Policy Checklist.

Statistics

For all statistical analyses, confirm that the following items are present in the figure legend, table legend, main text, or Methods section.

n/a	Confirmed	
☐	☒	The exact sample size (*n*) for each experimental group/condition, given as a discrete number and unit of measurement
☐	☒	A statement on whether measurements were taken from distinct samples or whether the same sample was measured repeatedly
☐	☒	The statistical test(s) used AND whether they are one- or two-sided<br>*Only common tests should be described solely by name; describe more complex techniques in the Methods section.*
☒	☐	A description of all covariates tested
☐	☒	A description of any assumptions or corrections, such as tests of normality and adjustment for multiple comparisons
☐	☒	A full description of the statistical parameters including central tendency (e.g. means) or other basic estimates (e.g. regression coefficient) AND variation (e.g. standard deviation) or associated estimates of uncertainty (e.g. confidence intervals)
☐	☒	For null hypothesis testing, the test statistic (e.g. *F*, *t*, *r*) with confidence intervals, effect sizes, degrees of freedom and *P* value noted<br>*Give P values as exact values whenever suitable.*
☒	☐	For Bayesian analysis, information on the choice of priors and Markov chain Monte Carlo settings
☒	☐	For hierarchical and complex designs, identification of the appropriate level for tests and full reporting of outcomes
☒	☐	Estimates of effect sizes (e.g. Cohen's *d*, Pearson's *r*), indicating how they were calculated

Our web collection on statistics for biologists contains articles on many of the points above.

Software and code

Policy information about availability of computer code

Data collection	Ultraviolet-visible absorption spectra (UV-vis) and fluorescence emission spectra (FL) were recorded on a Shimadzu UV-vis UV-2600 spectrometer and Hitachi F7000 spectrometer, respectively. Confocal imaging data collected using FV500 (Olympus).
Data analysis	Confocal imaging data analyzed using ImageJ 1.53a; Data process was conducted using Origin 9.0.

For manuscripts utilizing custom algorithms or software that are central to the research but not yet described in published literature, software must be made available to editors and reviewers. We strongly encourage code deposition in a community repository (e.g. GitHub). See the Nature Research guidelines for submitting code & software for further information.

Data

Policy information about availability of data

All manuscripts must include a data availability statement. This statement should provide the following information, where applicable:

- Accession codes, unique identifiers, or web links for publicly available datasets
- A list of figures that have associated raw data
- A description of any restrictions on data availability

The authors declare that all data generated in this study are provided in the article and the Supplementary Information file, and are also available from the corresponding author upon request. Source data are provided with this paper.

April 2020

Field-specific reporting

Please select the one below that is the best fit for your research. If you are not sure, read the appropriate sections before making your selection.

☒ Life sciences ☐ Behavioural & social sciences ☐ Ecological, evolutionary & environmental sciences

For a reference copy of the document with all sections, see nature.com/documents/nr-reporting-summary-flat.pdf

Life sciences study design

All studies must disclose on these points even when the disclosure is negative.

Sample size	Number of technical replicates and biological replicates are reported in the figure legends. Sample size was determined based on variability of the response deviating from the mean as indicated by the standard error of the mean, which is also represented in the figures. Typically, data are represented as mean values and first standard deviations determined from more than three experiments.
Data exclusions	N/A
Replication	In case of tubular prototissue vessel construction and nitric oxide generation, experiments were replicated on independent days for three times. In case of anticoagulation activity in prototissue vessels, experiments were performed on different rabbits for three times. Replication attempts were successful.
Randomization	The samples were allocated into experiment randomly.
Blinding	Not applicable for in vitro experiments.

Reporting for specific materials, systems and methods

We require information from authors about some types of materials, experimental systems and methods used in many studies. Here, indicate whether each material, system or method listed is relevant to your study. If you are not sure if a list item applies to your research, read the appropriate section before selecting a response.

Materials & experimental systems

n/a	Involved in the study	
☒	☐	Antibodies
☒	☐	Eukaryotic cell lines
☒	☐	Palaeontology and archaeology
☐	☒	Animals and other organisms
☒	☐	Human research participants
☒	☐	Clinical data
☒	☐	Dual use research of concern

Methods

n/a	Involved in the study	
☒	☐	ChIP-seq
☒	☐	Flow cytometry
☒	☐	MRI-based neuroimaging

Animals and other organisms

Policy information about studies involving animals; ARRIVE guidelines recommended for reporting animal research

Laboratory animals	New Zealand white rabbits (8 months old, either sex) weight from 2.0-2.5 kg were used .
Wild animals	N/A
Field-collected samples	N/A
Ethics oversight	All procedures with the animal experiments in this study were performed in accordance with the guidelines of the National Institutes of Health for the Care and Use of Laboratory Animals and were approved by the Ethics Committee on Animal Care of Hunan University (No. SYXK (Xiang) 2018-0006).

Note that full information on the approval of the study protocol must also be provided in the manuscript.
